# Structural and Organizational Strategies of Locomotor Modules during Landing in Patients with Chronic Ankle Instability

**DOI:** 10.3390/bioengineering11050518

**Published:** 2024-05-20

**Authors:** Tianle Jie, Datao Xu, Zanni Zhang, Ee-Chon Teo, Julien S. Baker, Huiyu Zhou, Yaodong Gu

**Affiliations:** 1 Faculty of Sports Science, Ningbo University, Ningbo 315211, China; 2 Faculty of Engineering, University of Pannonia, 8201 Veszprem, Hungary; 3 School of Chemical and Biomedical Engineering, Nanyang Technological University, Singapore 639798, Singapore; 4 Faculty of Engineering, University of Szeged, 6720 Szeged, Hungary

**Keywords:** muscle synergy, non-negative matrix factorization, muscle activation model, K-means clustering, chronic ankle instability

## Abstract

Background: Human locomotion involves the coordinated activation of a finite set of modules, known as muscle synergy, which represent the motor control strategy of the central nervous system. However, most prior studies have focused on isolated muscle activation, overlooking the modular organization of motor behavior. Therefore, to enhance comprehension of muscle coordination dynamics during multi-joint movements in chronic ankle instability (CAI), exploring muscle synergies during landing in CAI patients is imperative. Methods: A total of 22 patients with unilateral CAI and 22 healthy participants were recruited for this research. We employed a recursive model for second-order differential equations to process electromyographic (EMG) data after filtering preprocessing, generating the muscle activation matrix, which was subsequently inputted into the non-negative matrix factorization model for extraction of the muscle synergy. Muscle synergies were classified utilizing the K-means clustering algorithm and Pearson correlation coefficients. Statistical parameter mapping (SPM) was employed for temporal modular parameter analyses. Results: Four muscle synergies were identified in both the CAI and healthy groups. In Synergy 1, only the gluteus maximus showed significantly higher relative weight in CAI compared to healthy controls (*p* = 0.0035). Synergy 2 showed significantly higher relative weights for the vastus lateralis in the healthy group compared to CAI (*p* = 0.018), while in Synergy 4, CAI demonstrated significantly higher relative weights of the vastus lateralis compared to healthy controls (*p* = 0.030). Furthermore, in Synergy 2, the CAI group exhibited higher weights of the tibialis anterior compared to the healthy group (*p* = 0.042). Conclusions: The study suggested that patients with CAI exhibit a comparable modular organizational framework to the healthy group. Investigation of amplitude adjustments within the synergy spatial module shed light on the adaptive strategies employed by the tibialis anterior and gluteus maximus muscles to optimize control strategies during landing in patients with CAI. Variances in the muscle-specific weights of the vastus lateralis across movement modules reveal novel biomechanical adaptations in CAI, offering valuable insights for refining rehabilitation protocols.

## 1. Introduction

Lateral ankle sprains represent a prevalent musculoskeletal injury, accounting for approximately 10–30% of all sports-related injuries [1]. This condition impacts a substantial proportion of the physically active population, leading to complications such as osteoarthritis, sensory limb dysfunction, notable decline in quality of life, and significant life burdens [2]. According to the International Ankle Consortium, it is estimated that about 70% of individuals who lack timely and effective management and treatment following an ankle sprain invariably progress to chronic ankle instability (CAI) [3]. Patients with CAI typically exhibit recurrent ankle sprains, compromised support stability, frequent episodes of the ankle giving way, and biomechanical abnormalities [4]. In their recent review, Jay et al. provide insights into the latest advancements in ankle sprain modeling [5]. Efforts aimed at enhancing rehabilitation strategies for patients with CAI have transitioned from mere anatomical interventions to a dynamic integration of mechanical and sensorimotor injury considerations.

Numerous studies have explored perceptual dysfunction in individuals with CAI, a phenomenon closely linked to physiological alterations resulting from ligament injuries [6,7]. Specifically, these injuries correlate with reduced activation of gamma motor neurons and attenuated sensitivity of muscle spindles, ultimately resulting in a deterioration of the patient’s ability to perceive mechanical stimuli (i.e., force and vibration), as well as proprioceptive feedback relating to joint position and motion, consequently affecting ankle stability [8,9]. A comprehensive motor control process involves the reception of external stimuli by receptors, the generation of control signals by the brain, and the stimulation of muscle contraction by motor neurons situated in the spinal cord [10,11]. A reduction in perceptual function among CAI patients inevitably disrupts signal transmission, resulting in aberrant muscle activation, subsequently leading to abnormal motor behavior [12]. Abnormal muscle activation patterns involving the peroneus longus, tibialis anterior, and medial gastrocnemius muscles have been consistently observed in various studies [13,14]. These findings indicate that individuals with CAI typically employ a multi-joint or multi-muscle strategy to maintain stability, suggesting that positive adaptations may slightly alter locomotion patterns in CAI patients to mitigate the effects of sprains. However, there remains a lack of clear elucidation regarding how the nervous system coordinates the activation of multiple muscles to achieve adaptive movements.

Furthermore, individuals with CAI demonstrate specific adaptive alterations in kinematics compared to healthy populations. Studies have revealed that CAI patients exhibit a smaller ankle plantarflexion angle and an increased hip flexion angle at initial contact during landing [15,16]. The decrease in ankle plantarflexion angle can effectively mitigate the tension on the lateral ankle ligaments, thereby positioning the ankle joint in a more stable position and reducing the risk of injury associated with the plantarflexion inversion position [17]. Researchers suggest that this adaptive response may represent a reflexive self-protective mechanism exhibited by CAI patients with ankle sprains [15]. Additionally, an increase in hip flexion angle was observed in CAI, with this movement pattern prevalent among the majority of individuals with CAI during landing. This adaptation can be attributed to ankle injuries prompting adaptive alterations in the central nervous system, thereby influencing centrally-controlled motor control. Consequently, neuromuscular deficits manifest in the proximal joints of the lower extremity on the injured side [18,19]. These varied motor control strategies involve different joints and muscles, all with the unified objective of redistributing redundant structures to mitigate the functional decline or loss induced by ankle sprains across various patterns.

Researchers commonly utilize electromyography (EMG) to identify neuromuscular control strategies in specific tasks and delve into the underlying mechanisms of altered motor control strategies. However, EMG typically focuses solely on monitoring and analyzing the electrical activity of individual muscles, thereby offering somewhat limited insights [20]. Most muscles operate as functional units within a given task, guided by their anatomical structure and neural circuitry [21]. In essence, a single muscle may contribute to multiple movement patterns, while a locomotion pattern may involve coordinated activity of multiple muscles [22]. To accurately identify neuromuscular control strategies in each given task and to acquire comprehensive insights, it is imperative to consider the synergistic interaction of multiple muscles in an integrated manner. 

Muscle synergy serves as a fundamental aspect of modular control in human movement [23]. A limited number of modules coordinates the activation of diverse muscle groups with varying weights to accomplish specific tasks, all under the regulatory oversight of the nervous system [24]. The extraction process of muscle synergy is fundamentally a matrix decomposition method, commonly utilizing algorithms such as Principal Component Analysis (PCA), Factor Analysis (FA), Non-negative Matrix Factorization (NNMF), Independent Component Analysis (ICA), Autoencoder models, Hierarchical Alternating Least Squares (HALS) algorithm, and Multivariate Curve Resolution-Alternating Least Squares (MCR-ALS). By inputting muscle activation matrices into these algorithms, we can obtain synergy modules that are invariant over time and activation coefficients associated with specific tasks. Non-negative matrix factorization (NNMF) algorithms [25], characterized by their imposition of non-negativity constraints on the original data matrix and yielding muscle synergies closely matched to the probability distributions of muscle activation patterns during dynamic tasks, are extensively employed for extracting muscle synergy patterns [26]. Employing the NNMF algorithm, Kim et al. explored muscle synergy patterns in CAI patients during both anticipated and unanticipated cutting [27]. Their findings indicated that while CAI patients and the healthy control group shared muscle synergy patterns, disparities in the weighting of the tibialis anterior within the ankle joint strategy may provide crucial insights for rehabilitating neuromuscular deficits in CAI patients. The landing maneuver represents a high-risk activity associated with ankle sprains [28]. Nevertheless, to our knowledge, there is a dearth of studies investigating muscle synergy patterns during landing in individuals with CAI. 

Therefore, the objective of this study was to investigate and compare muscle synergy patterns during landing in individuals with and without CAI. Based on previous studies, it is plausible to hypothesize that the number of muscle synergies remains unchanged in individuals with CAI. However, alterations in muscle-specific weights and activation timings are anticipated to occur, serving to accommodate the novel biomechanical demands imposed by ankle sprains.

## 2. Materials and Methods

**Figure 1:**
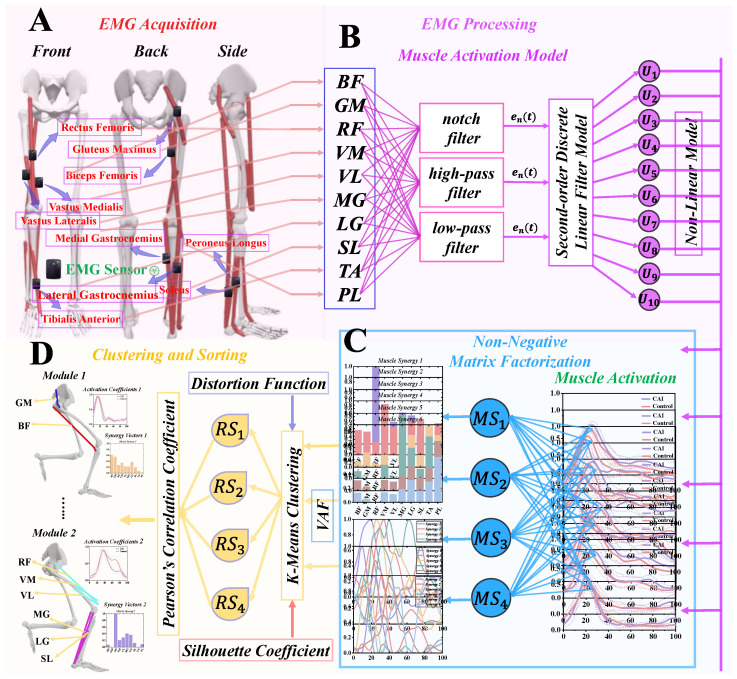
Overview of the overall workflow of the current study. (**A**) Acquisition of sEMG. (**B**) sEMG from 10 muscles were utilized to derive muscle activation using a muscle activation model. (**C**) Muscle synergy extraction using the NNMF algorithm. (**D**) Sorting of muscle synergies using the K-means clustering algorithm and Pearson’s correlation coefficient.

The overall framework of this study encompasses four primary components: (1) collection of surface electromyographic (sEMG) signals from the lower limb muscles; (2) estimation of muscle activation through a second-order discrete linear filter model; (3) identification of muscle synergy patterns using the NNMF algorithm; and (4) categorization of muscle synergy utilizing the K-means clustering algorithm and Pearson’s correlation coefficient. As illustrated in Figure 1, the experimental procedure involved several key steps. Firstly, sEMG signals from 10 lower limb muscles were acquired using EMG sensors during dynamic tasks performed by the subjects (Figure 1A). Secondly, the acquired EMG signals underwent filter preprocessing and were inputted into a second-order discrete linear filter model to recursively estimate neural activation. Subsequently, the muscle activation was determined by applying the estimated neural activation to a nonlinear model (Figure 1B). Thirdly, the muscle synergy matrix was derived by decomposing the obtained muscle activation matrix using the NNMF model (Figure 1C). Lastly, muscle synergy was classified using the K-means clustering algorithm and Pearson’s correlation coefficient, resulting in the identification of four motor modules (Figure 1D).

### 2.1. Participants

A total of 22 patients with unilateral CAI and 22 healthy participants were recruited for this research (Table 1). Initial screening and exclusion were performed based on the consensus statement of the International Ankle Consortium. Patients with CAI were defined as individuals meeting the following criteria: (1) CAIT score ≤ 24; (2) a record of traumatic ankle sprains necessitating two or more medical consultations; (3) reporting recurrent lateral ankle sprains lasting at least six months or expressing concern about potential ankle dysfunction. Individuals without history of lateral ankle sprains and a CAIT score of ≥29 were included in the healthy control group. Participants were excluded if they had a history of lower extremity surgery, lower extremity fractures, or bilateral ankle sprains. To mitigate the influence of limb dominance on the experiment, all participants included in the study had their right limb designated as the dominant limb, including the injured limb. The dominant limb was identified as the preferred leg for kicking a ball.

All participants were informed of the purpose, requirements, and procedures of the study before signing written informed consent. The study protocol was approved by the Scientific Research Ethics Committee of Ningbo University (Approval Number: RAGH20231120).

### 2.2. Experimental Protocol and Data Recordings

Following SENIAM guidelines to place the EMG sensors, during sEMG data collection, meticulous attention was given to selecting the optimal position on the muscle belly, avoiding areas near tendons or muscle edges. Additionally, to ensure consistent and accurate signal capture, electrodes were affixed parallel to the subject’s skin surface using biocompatible adhesive tape. Prior to electrode placement, the skin surface was meticulously cleaned with alcohol to remove any residual oils, debris, or hair. Ten EMG sensors (Delsys, Boston, MA, USA) were used to quantify muscle activations by positioning over the muscle bellies of the soleus (SL), medial gastrocnemius (MG), lateral gastrocnemius (LG), tibialis anterior (TA), peroneus longus (PL), rectus femoris (RF), vastus medialis (VM), vastus lateralis (VL), biceps femoris (BF), and gluteus maximus (GM).

Prior to starting the formal experiment, all participants were given a 15-min window to perform a dynamic warm-up and practice a single-leg jump landing to order to familiarize themselves with the task. They practiced without receiving feedback or specific instructions on landing technique to minimize any potential influence on their natural landing patterns. During the single-leg jump landing task, participants were instructed to stand on their injured leg, positioning themselves at a distance approximately equal to one leg’s length (measured from the greater trochanter to the lateral malleolus) from the designated endpoint [29]. They were also directed to place their hands on both sides of their hips, with the goal of minimizing the influence of arm swing on the experimental procedure. Following that, they had to jump over a hurdle with a height of 15 cm, landing on the same leg. They were then instructed to promptly stabilize and maintain balance for 5 s, while keeping their gaze forward.

All trials were conducted barefoot, with each participant performing three successful trials of single-leg jump landings. A trial was considered successful if participants landed squarely on the designated endpoint with their entire foot, while trials where participants struggled to maintain balance or exhibited extraneous movements were classified as unsuccessful. The experimental protocol continued until a cumulative total of three successful trials were achieved, with three-minute intervals separating each trial. Additionally, EMG signals were recorded during maximal voluntary contractions (MVC) for normalization of muscle activation. The detailed procedure for obtaining MVC data is provided in Supplementary Text S1. EMG data were acquired utilizing an EMGworks Acquisition measurement system (Delsys, Boston, MA, USA) and sampled at a rate of 1000 Hz.

### 2.3. Muscle Activation Model

The muscle activation model was built to precisely depict the temporal dynamics of muscle activation. A custom MATLAB (MathWorks, Inc., Natick, MA, USA) script was utilized to construct the muscle activation model from the raw sEMG. 

Initially, the raw sEMG underwent preprocessing, comprising three main steps: (a) applying a 50 Hz notch filter to eliminate industrial frequency interferences; (b) employing a 30 Hz zero-phase high-pass filter to eliminate motion artifacts; (c) conducting full-wave rectification. Subsequently, the sEMG signal underwent 5 Hz zero-phase low-pass filter, aiming to emulate muscles’ low-pass filter characteristics. The sEMG during MVC underwent the same processing method. The peak value of the sEMG during MVC was set as 100% muscle activation, followed by normalizing the EMG signal by this peak value to derive the normalized EMG signal $e_{n}(t)$. 

Traditional muscle activation models inadequately capture the dynamic relationship between neural signals and muscle force output, potentially introducing bias in interpreting muscle activation [30,31]. This study employed a second-order discrete linear filter model to characterize the muscle activation $a_{n}(t)$ from the normalized EMG signal $e_{n}(t)$, while solving neural activation $u_{n}(t)$ recursively:

(1) $$u_{n}\left(t\right)=\alpha e_{n}\left(t-d\right)-\beta_{1}u_{n}\left(t-1\right)-\beta_{2}u_{n}\left(t-2\right)$$

where t represents the time sequence of activation of the normalized EMG signal $e_{n}(t)$. The neural activation $u_{n}(t)$ is influenced by the preceding two neural activations, $u_{n}\left(t-1\right)$ and $u_{n}\left(t-2\right)$. The electro-mechanical delay d typically set to 10 ms, indicating the time delay between neural activation $u_{n}(t)$ and muscle contraction. Additionally, parameter α represents the gain coefficient, modulating the relationship between the activation signal $e_{n}(t)$ and the electrophysiological signal of muscle n:

(2) $$\alpha -\beta_{1}-\beta_{2}=1$$

To ensure convergence to the correct solution under specific conditions, we define $C_{1}$ and $C_{2}$ as constant coefficients representing constraints, allowing $\beta_{1}$ and $\beta_{2}$ to be expressed as follows:

(3) $$\beta_{1}=C_{1}+C_{2},\left|C_{1}\right|<1$$

(4) $$\beta_{2}=C_{1}\times C_{2},\left|C_{2}\right|<1$$

**Figure 2:**
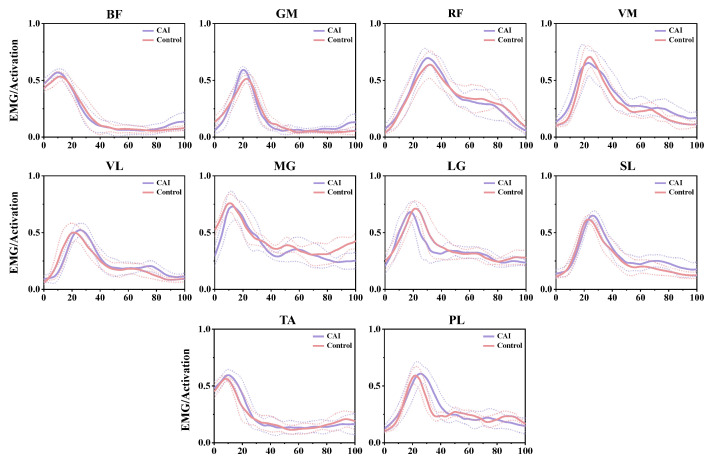
Muscle activation profiles during landing in the CAI and healthy groups following muscle activation model processing.

Muscle activation $a_{n}(t)$ is subsequently determined based on neural activation $u_{n}(t)$ using a non-linear model (Figure 2):

(5) $$a_{n}\left(t\right)=\frac{e^{A_{n}u_{n}(t)}-1}{e^{A_{n}}-1}$$

The relationship between neural activation $u_{n}(t)$ and muscle activation $a_{n}(t)$ is typically nonlinear [32]. Thus, we incorporate the nonlinear shape factor $A_{n}$ as a constraint. In this study, $A_{n}$ was set to 1.5 [33,34].

### 2.4. Non-Negative Matrix Factorization Extracts Muscle Synergies

The non-negative matrix factorization (NNMF) algorithm was applied to extract muscle synergies from the data matrix of EMG processed by the muscle activation model for each subject and condition [25]. The EMG data matrix V comprises m × n×repetitionsoftrials, where m represents the number of muscles, and n corresponds to the number of data point. NNMF is a dimensionality reduction technique based on a low-rank approximation of the feature space [35]. This factorization technique enables the decomposition of the EMG data matrix into a linear combination of two low-dimensional components: the synergy vectors W (m×k matrix, where k is the number of muscle synergies, k<m, $W\in R_{+}^{m\times k}$) and the activation coefficients C (k×n matrix, $C\in R_{+}^{k\times n}$). NNMF can be mathematically expressed as follows:

(6) $$V_{\left(m\times n\right)}=W_{\left(m\times k\right)}\times C_{\left(k\times n\right)}+\varepsilon_{\left(m\times n\right)}$$

where ε is the residual error matrix, and NNMF permits certain reconstruction errors. Synergy vector W represents the relative contribution of individual muscles in organizing the corresponding motor module, while C represents the change in the temporal order of the synergy vectors W modulated by the central nervous system.

The NNMF solution represents an optimization problem aimed at approximating W × C to V, minimizing error term ε as much as possible. This optimal solution can be expressed as:

(7) $${min}_{W,C}\left\|V-W\times C\right\|$$

To solve Equation (7), when ε follows a Gaussian distribution, we utilize the Euclidean distance between the decomposed matrix W×C and the EMG data matrix V as the cost function to quantify the accuracy of the decomposition, expressed as:

(8) $$∥V-WC{∥}^{2}=\sum_{m,n}(V_{mn}-(WC{)}_{mn}{)}^{2}$$

The solution of the cost function is derived through an algorithm based on the gradient descent method, utilizing the multiplicative update rules to find the locally optimal solution [36]. According to the additive update rule, W and C are updated as follows:

(9) $$W_{\left(m\times k\right)}=W_{\left(m\times k\right)}-\gamma_{mk}\times \left[{\left({VC}^{T}\right)}_{\left(m\times k\right)}-{\left(WCC^{T}\right)}_{\left(m\times k\right)}\right]$$

(10) $$C_{\left(k\times n\right)}=C_{\left(k\times n\right)}-\eta_{kn}\times \left[{\left(W^{T}V\right)}_{\left(k\times n\right)}-{\left(WCW^{T}\right)}_{\left(k\times n\right)}\right]$$

where $\gamma_{mk}$ = $\frac{W_{\left(m\times k\right)}}{{\left(WCC^{T}\right)}_{\left(n\times k\right)}}$ and $\eta_{kn}=\frac{C_{\left(k\times n\right)}}{{\left(WCW^{T}\right)}_{\left(k\times n\right)}}$ are the learning rates, assigning $\gamma_{mk}$ and $\eta_{kn}$ to Equations (9) and (10), ultimately obtaining:

(11) $$W_{\left(m\times k\right)}=W_{\left(m\times k\right)}\times \frac{{\left({VC}^{T}\right)}_{\left(m\times k\right)}}{{\left(WCC^{T}\right)}_{\left(m\times k\right)}}$$

(12) $$C_{\left(k\times n\right)}=C_{\left(k\times n\right)}\times \frac{{\left(W^{T}V\right)}_{\left(k\times n\right)}}{{\left(WCW^{T}\right)}_{\left(k\times n\right)}}$$

The algorithm update steps are as follows: (a) randomly generate a matrix W; (b) fix W and iteratively update C according to Equation (11) until convergence; (c) fix C and iteratively update W according to Equation (12) until convergence; (d) repeat steps b and c until the corresponding cost function remains unchanged or changes very little.

The Variance Accounted For (VAF) was utilized to identify the optimal number of synergies, indicating the degree to which the decomposition accurately reconstructs the EMG data matrix $V_{r}$. The VAF can be expressed as:

(13) $${VAF}_{global}=1-\frac{\sum_{m,n}{\left(V-V_{r}\right)}_{m,n}^{2}}{\sum_{m,n}V_{m,n}^{2}}$$

(14) $${VAF}_{muscle}=1-\frac{\sum_{n}{\left(V-V_{r}\right)}_{m_{each}n}^{2}}{\sum_{n}V_{m_{each}n}^{2}}$$

where $m_{each}$ represents each muscle. The optimal number of synergies $N_{opt}$ is determined according to the following protocol [37]: We iteratively increase the parameter k, where k represents positive integers ranging from 1 to m, while concurrently calculating both the global VAF and local muscle VAF. Once the global VAF > 90% and simultaneously the local muscle VAF > 75% for the first time, we designate the corresponding k value as the optimal number of synergies.

### 2.5. K-means Clustering Algorithm for Sorting Muscle Synergies

In this study, the K-means clustering algorithm was used for the classification of muscle synergies. Specifically, it integrated the matrix of synergy vectors W decomposed for all healthy participants across various conditions, into a matrix $D_{\left({m\times k}_{total}\right)}$ for subsequent clustering analysis [38]. The concept behind the K-means clustering algorithm is to minimize the intra-cluster distances while maximizing the inter-cluster distances, ensuring that data within clusters are closely grouped while maintaining maximum separation between clusters [39].

The protocol for classifying muscle synergies using the K-means clustering algorithm follows: (a) in the Unlabeled datasets $x^{\left(1\right)},x^{\left(2\right)},\ldots ,x^{\left(k_{total}\right)}$, i cluster centroids $\mu_{i}(i\in 1,2,\ldots ,n)$ are randomly selected, equivalent to the existence of i clusters $c^{\left(i\right)}$; (b) for each $x^{\left(j\right)}(j\in [1,k_{total}])$, its distance to each $\mu_{i}$ needs to be computed, assigning $x^{\left(j\right)}$ to the cluster $c^{\left(i\right)}$ with the nearest $\mu_{i}$ to it. This process is mathematically expressed as:

(15) $$c^{\left(i\right)}:=argmin_{i}||x^{\left(j\right)}-\mu_{i}|{|}^{2}$$

(c) recalculate the cluster centroids $\mu_{i}$ for each cluster $c^{\left(i\right)}$:

(16) $$\mu_{i}=\frac{\sum_{j=1}^{k_{total}}l\{c^{\left(j\right)}=i\}x^{\left(j\right)}}{\sum_{j=1}^{k_{total}}l\{c^{\left(j\right)}=i\}}$$

(d) repeat steps b and c until the algorithm converges. To ensure complete convergence of the algorithm, the distortion function is introduced here [40]:

(17) $$J(c,\mu )=\sum_{i=1}^{m}||x^{\left(j\right)}-\mu_{c^{\left(j\right)}}|{|}^{2}$$

If J(c,μ) does not reach its minimum value, the cluster centroids $\mu_{i}$ for each cluster can be updated while keeping $c^{\left(i\right)}$ fixed. As the cluster centroids change, the corresponding cluster assignments also change and the process iterates continuously. When J(c,μ) reaches its minimum value, both $\mu_{i}$ and $c^{\left(i\right)}$ converge simultaneously. The quality of the clustering was evaluated using the Silhouette Coefficient, which was computed as follows:

(18) $$S(i)=\frac{b(i)-a(i)}{max(a(i),b(i))}$$

Suppose $x^{\left(j\right)}$ belongs to cluster $c^{\left(i\right)}$, where a(i) represents the average distance of $x^{\left(j\right)}$ from other data in the same cluster, and b(i) represents the average distance of $x^{\left(j\right)}$ from all data in the nearest cluster $c^{\left(i\right)}$.

The cluster centroids $u^{\left(i\right)}$ formed by clustering the synergy vectors W are used as the baseline reference synergy. Subsequently, we categorized all synergy vectors of each subject under each condition separately using Pearson’s correlation coefficient [41]. This was achieved by computing correlation coefficients (r) between the subjects’ synergy vectors and the cluster centroids $u^{\left(i\right)}$. If r > 0.6, we regarded the two synergies as similar and grouped them together. Upon completing the classification of synergy vectors W, the corresponding activation coefficients C were automatically assigned to the same category.

### 2.6. Outcome Variables

The outcome variables of interest include the synergy vectors and activation coefficients for each group. Specifically, the parameters of interest for the synergy vectors are the number of synergy patterns $N_{syn}$, and the weight of the synergy vector corresponding to each muscle $I_{muscle}$. Regarding the activation coefficients, the parameters of interest include the temporal dynamics captured by the waveform curves of the activation coefficient time series. To mitigate potential variations in gait cycles or movement durations among individuals or experiments, it was imperative to standardize the comparability of data from different experiments or individuals. This standardization aimed to facilitate statistical analyses and enhance the interpretability of results. For variables exhibiting temporal variability, data normalization to 101 points (corresponding to the 0–100% range of the landing phase) was conducted using customized MATLAB code.

### 2.7. Statistical Analysis

The normality and homogeneity of variances in the outcome variables were assessed using the Shapiro–Wilk test and Levene’s test, respectively. Independent sample t-tests were performed for subsequent data analysis if the outcome variables satisfied the assumptions of normal distribution and variance homogeneity. If the assumptions were violated, the non-parametric Wilcoxon rank-sum test was employed. The significance level was set at α = 0.05. Cohen’s d was employed to quantify the difference in magnitude between the two groups (CAI and healthy) and to assess the practical significance of the outcome variables:

(19) $$d=\frac{{\bar{X}}_{1}-{\bar{X}}_{2}}{s_{p}}$$

Effect sizes were interpreted based on established criteria: less than 0.2 indicated a small effect; between 0.2 and 0.5 indicated a medium effect; and greater than 0.8 indicated a large effect.

For a more comprehensive insight into the temporal changes in activation coefficients, we performed Statistical Parametric Mapping (SPM) analysis to compare activation coefficients across various time points [42]. The SPM also included calculating the critical threshold based on random field theory and generating SPM{T} curves. If SPM{T} exceeded the critical threshold, it indicated a significant difference in activation coefficients between groups at that particular time point. MATLAB (MathWorks, MA, USA) was used for statistical analyses.

## 3. Results

### 3.1. Number of Muscle Synergy Extraction by NNMF

**Figure 3:**
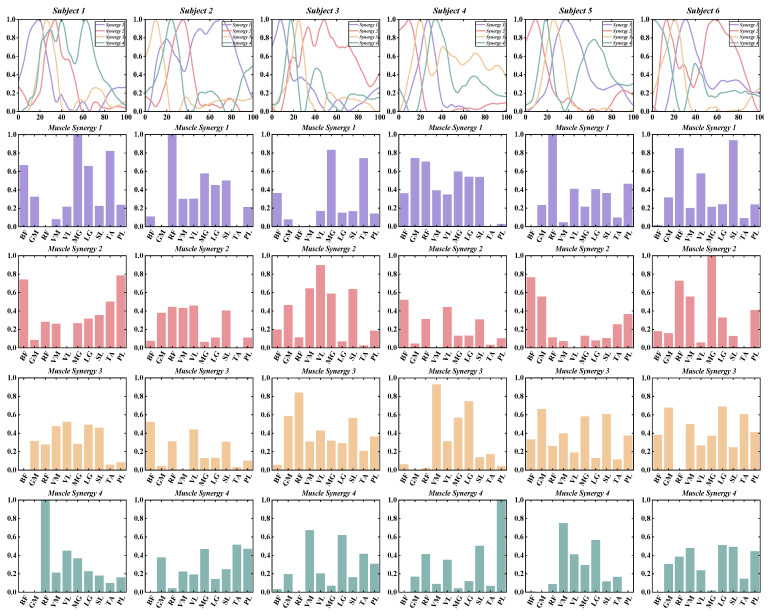
The extracted synergy vectors matrix and activation coefficient matrix from one to six subjects in the CAI group are presented, with activation coefficient results displayed in rows 1 and the remaining rows depicting the synergy vectors.

**Figure 4:**
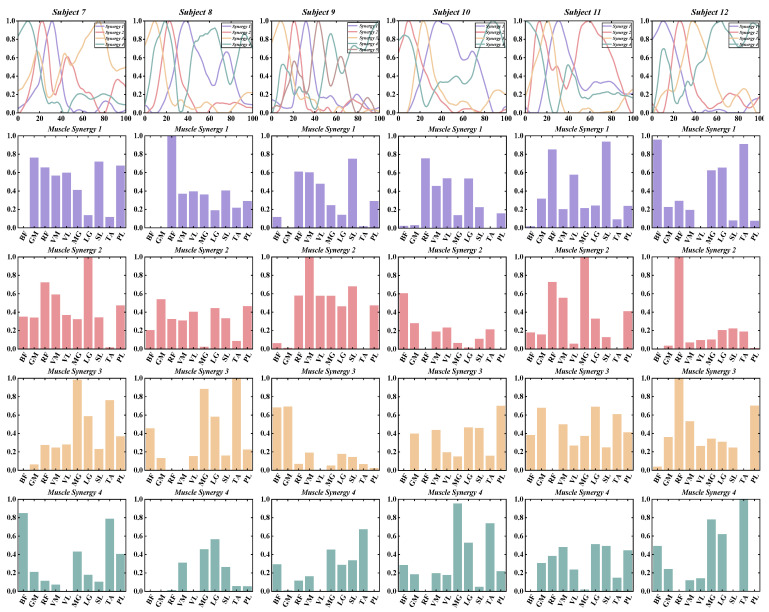
The extracted synergy vectors matrix and activation coefficient matrix from seven to 12 subjects in the CAI group are presented, with activation coefficient results displayed in rows 1 and the remaining rows depicting the synergy vectors.

**Figure 5:**
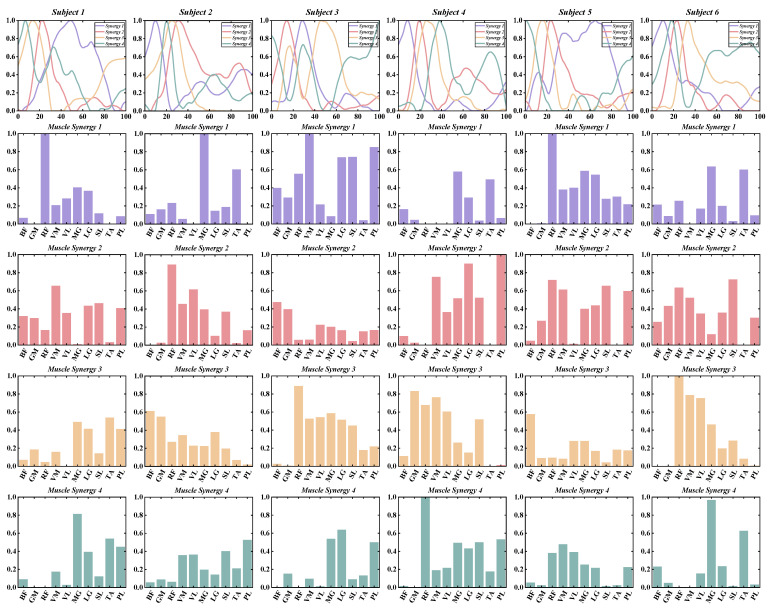
The extracted synergy vectors matrix and activation coefficient matrix from one to six subjects in the healthy group are presented, with activation coefficient results displayed in rows 1 and the remaining rows depicting the synergy vectors.

**Figure 6:**
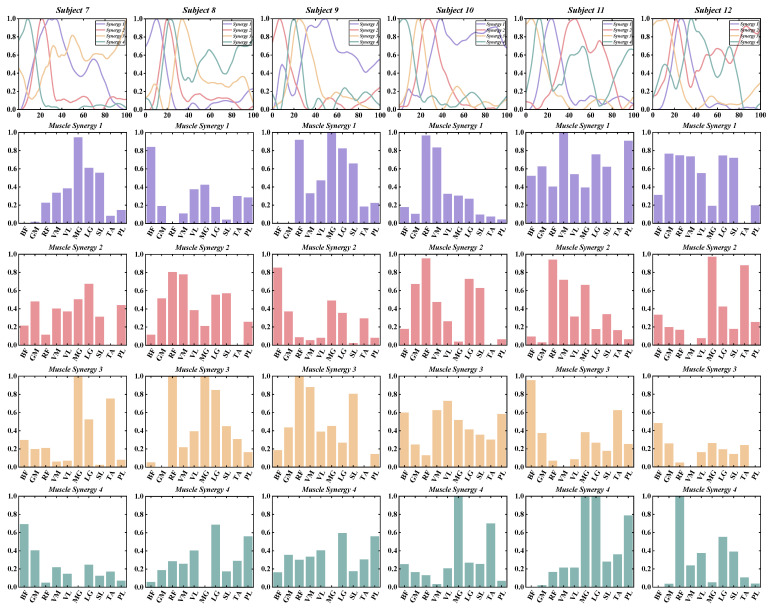
The extracted synergy vectors matrix and activation coefficient matrix from seven to 12 subjects in the healthy group are presented, with activation coefficient results displayed in rows 1 and the remaining rows depicting the synergy vectors.

According to predefined criteria for the optimal number of muscle synergies for each group in various conditions, we observed that the neuromotor organization of most CAI patients and healthy controls during landing could be characterized by four to six muscle synergy patterns. To enhance clarity in our presentation, we depict the synergy vectors matrix and activation coefficient matrix for only the four extracted muscle synergies from each subject group (Figure 3, Figure 4, Figure 5 and Figure 6). For a comprehensive decomposition of the resulting synergy vectors matrix and activation coefficients matrix, please refer to Supplementary Text S2.

**Figure 7:**
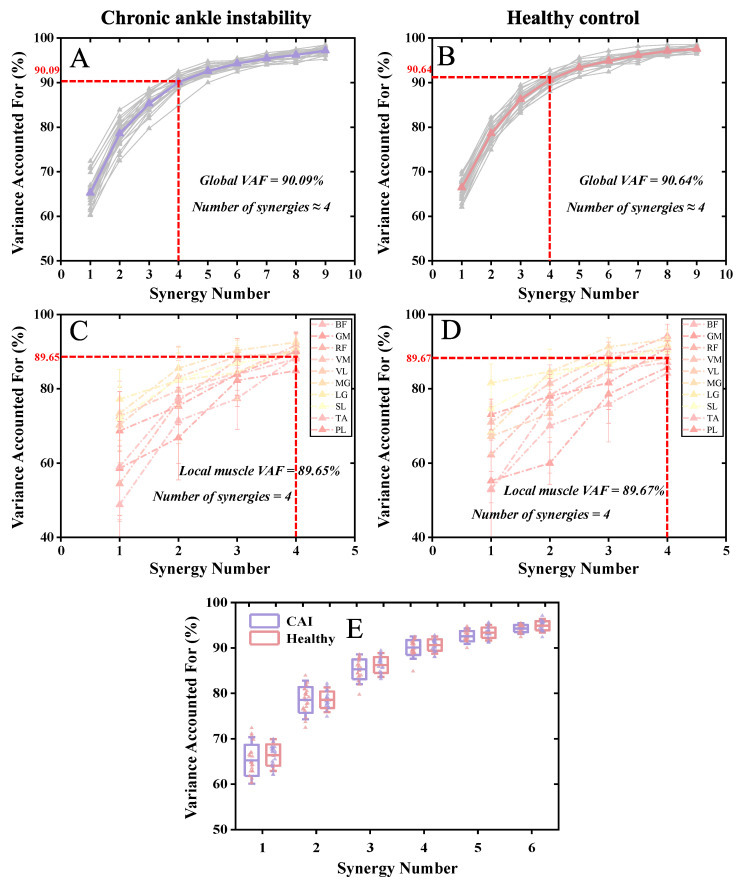
Global and local muscle VAFs. (**A**) The global VAF corresponding to each synergy in the CAI group. (**B**) The global VAF corresponding to each synergy in the healthy group. (**C**) The local muscle VAF corresponding to each synergy in the CAI group. (**D**) The local muscle VAF corresponding to each synergy in the healthy group. (**E**) Comparison of global VAF corresponding to muscle synergies 1–6 in the CAI and healthy groups.

In most cases, with four muscle synergies, both the CAI and healthy groups achieved global VAF above 90% and local muscle VAFs exceeding 75% (Table 2 and Figure 7).

No statistically significant differences were observed between the CAI and healthy groups regarding the number of muscle synergies, global VAF, and local muscle VAF (Table 2 and Figure 5).

### 3.2. Similarities between Muscle Synergies

**Figure 8:**
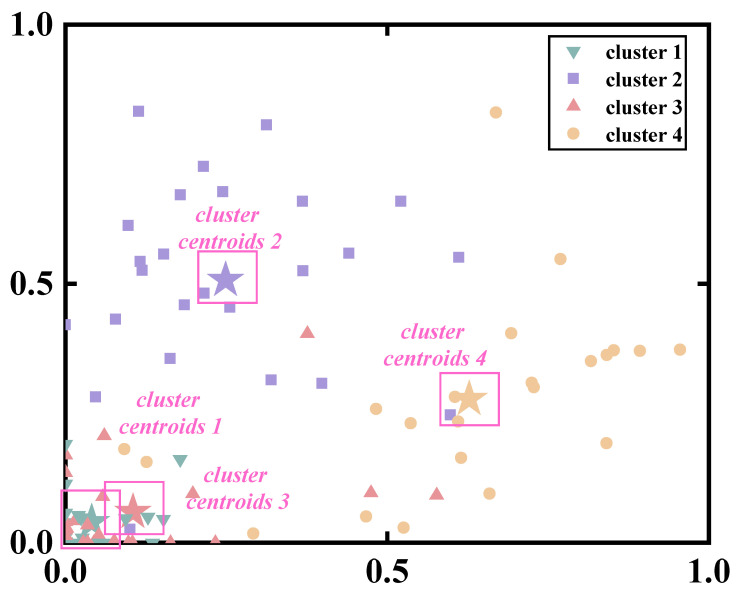
Visualizations of K-means clustering results for all synergy vectors in the healthy group.

By employing the Silhouette Coefficient for assessing clustering effects, we found that the clustering was optimal when i = 4, suggesting segmentation of datasets into four clusters (Figure 8). With increasing i, the Silhouette Coefficient decreased, indicating a deterioration in clustering quality.

**Figure 9:**
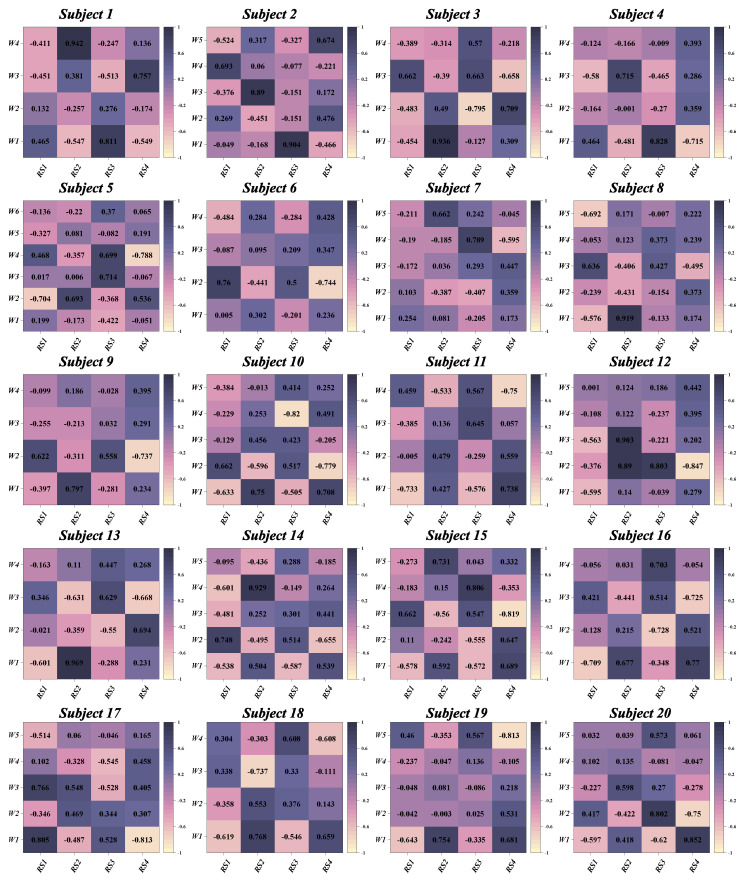
Correlation coefficients between synergy vectors and reference synergies for the 20 subjects in the CAI group. W: synergy vectors. RS: Reference Synergy.

**Figure 10:**
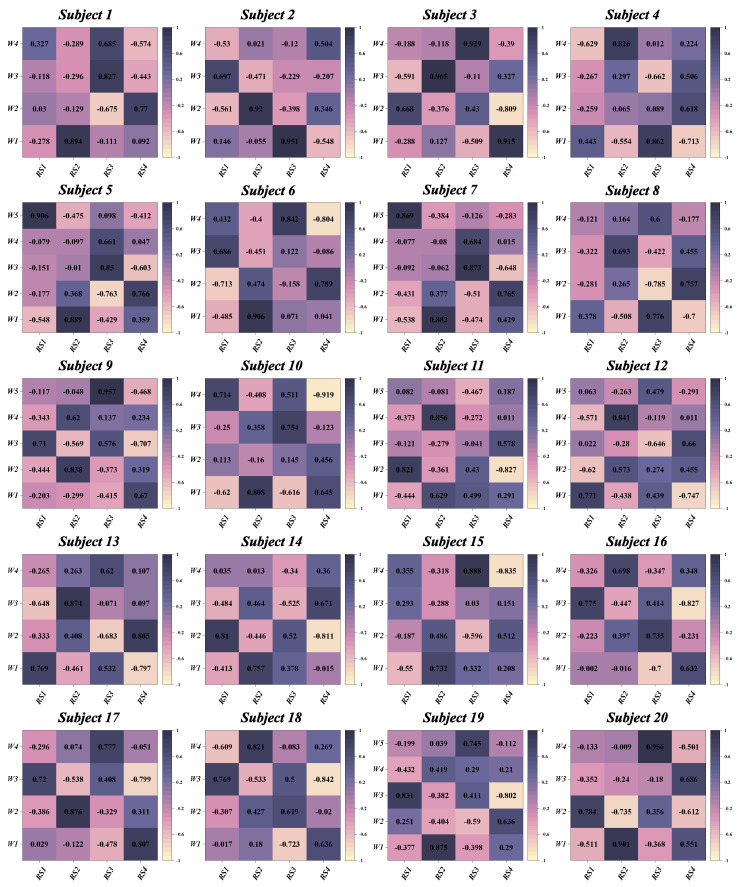
Correlation coefficients between synergy vectors and reference synergies for the 20 subjects in the healthy group. W: synergy vectors. RS: Reference Synergy.

Four cluster centroids were used as reference synergies and employed Pearson correlation coefficients to classify synergy patterns for each subject (Figure 9 and Figure 10). Additional comprehensive information regarding the correlation coefficients between the synergy vectors and the reference synergy for each participant in both groups can be found in Supplementary Text S3. Overall, we observed that the Healthy group exhibited greater similarity to the reference synergies compared to the CAI group (CAI: 52%, Healthy: 86.7%). Specifically, both the CAI and Healthy groups exhibited high similarity in Reference Synergy 2 (CAI: 32.7%, Healthy: 29.6%) and Reference Synergy 3 (CAI: 26.9%, Healthy: 28.4%).

### 3.3. The Functional Role of Muscle Synergies

**Figure 11:**
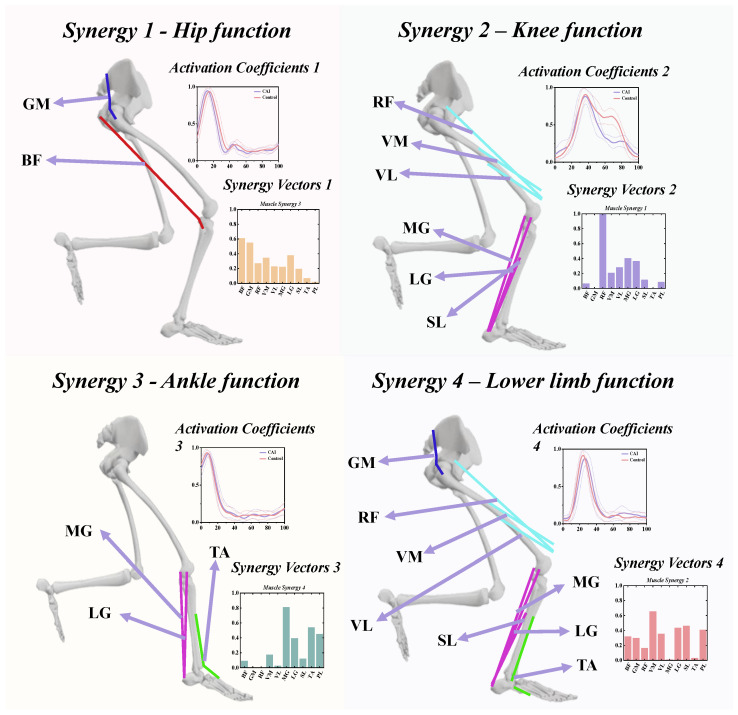
Visualization of four different movement modules corresponding to each of the four identified muscle synergies.

The extensive redundancy within muscle groups ensures robust joint adaptability and flexibility, yet they adhere to a constrained synergistic framework regulated by the central nervous system (CNS) to execute diverse motor tasks. To functionally interpret the specific mechanism of muscle synergy in patients with CAI, based on the muscle weight features in the decomposed low-dimensional elements, we specifically divide the motor control in landing into four functional modules (Figure 11).

Upon closer examination of each synergy, the muscle-specific weights in the synergy vector of Synergy 1 indicate that this synergy is primarily characterized by activation of the BF and GM, suggesting a synergy pattern that may correspond to hip function during landing. In Synergy 2, predominant activation was observed in the RF, VM, VL, MG, LG, and SL, indicating its activation throughout almost the entire landing duration, possibly contributing to knee stabilization. Synergy 3 primarily involved the MG, LG, and TA, with predominant activation occurring during 0–20% of the landing phase, suggesting a role in ankle control strategy during the initial contact phase. Regarding Synergy 4, the muscle-specific weights in the vector were associated with the activation of GM, RF, VM, VL, LG, SL, and PL, with most muscles active in this synergy pattern, indicating its responsibility for the overall lower limb motor control strategy.

### 3.4. Characteristics of Temporal and Spatial Modules

**Figure 12:**
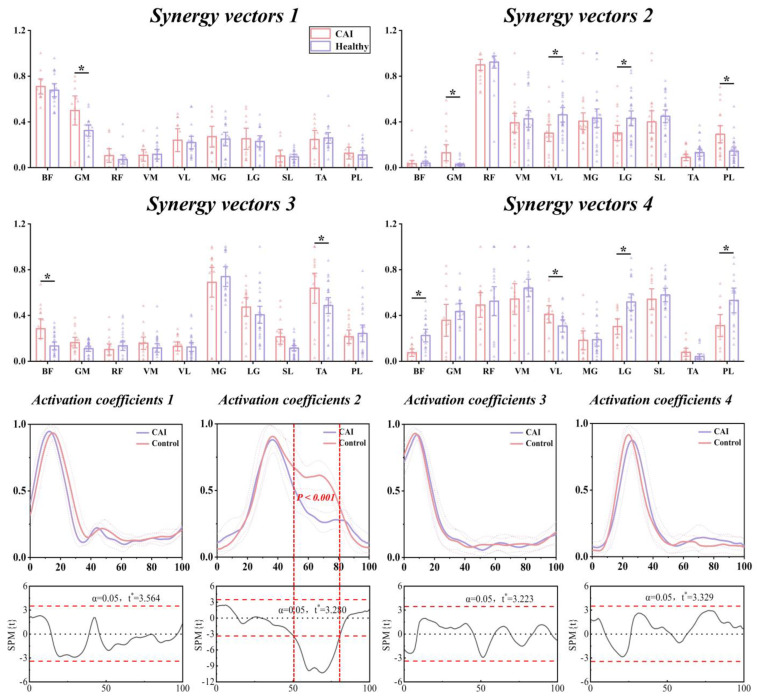
The synergy vectors, activation coefficients, and SPM1d results for activation coefficients in the muscle synergies extracted from each group. *: significant difference with *p* < 0.05.

In Synergy 1, there were no statistically significant differences in most of the muscle-specific relative weights, with only the GM observing a significantly higher relative weight (*p* = 0.0035, d = 0.903) in the CAI compared to the healthy group. As for Synergy 2, the relative weights of the GM and PL were significantly higher in CAI (*p* = 0.042, d = 0.852, *p* = 0.006, d = 0.910, respectively), while individuals in the healthy group showing significantly higher relative weights for the VL and LG (*p* = 0.018, d = 0.800, *p* = 0.049, d = 0.665, respectively), which are muscles that exhibited heightened activity within the corresponding motor module. Within Synergy 3, CAI exhibited significantly higher relative weights of the BF and the TA (*p* = 0.026, d = 0.929, *p* = 0.042, d = 0.595, respectively), while the BF displaying diminished contribution to the corresponding functional module and remaining inactive. For Synergy 4, CAI demonstrated significantly higher relative weights of the VL compared to the healthy group (*p* = 0.030, d = 0.873). Conversely, the relative weights of the BF, LG, and PL were significantly lower in CAI than in the healthy group (*p* = 0.002, d = 1.196, *p* = 0.005, d = 1.274, *p* = 0.047, d = 0.863, respectively). No significant differences in relative weights of other muscles were observed (Table 3 and Figure 12).

The SPM1d results revealed significant differences in activation coefficient only within Synergy 3, notably between 51% and 81% of the landing phase (*p* < 0.001), with significantly higher amplitude observed in CAI compared to healthy controls. Other muscle synergies exhibited comparable activation coefficient profiles (Figure 12).

## 4. Discussion

The objective of the present study was to examine muscle synergy patterns during landing in individuals with CAI. To the best of our knowledge, this study represents the first detailed quantification of the modular organizational strategy of neuromotor response in CAI patients during landing. Our study elucidates several key findings: (1) Individuals with CAI exhibited the specific muscle synergy patterns of the gluteus maximus during landing, highlighting its pivotal role in the modular control strategy of the central nervous system in this population. (2) Deficits were observed in coordinated multi-muscle control within the knee joint strategy of patients with CAI, evidenced by alterations in the spatial and temporal patterns of muscle synergy involving the vastus lateralis and lateral gastrocnemius. (3) In ankle control strategies, CAI patients showed a positive neuromotor response, characterized by efforts to increase tibialis anterior muscle contribution. (4) Patients with CAI may demonstrate adaptive responses to the altered biomechanical demands imposed by ankle sprains, manifesting through slight adjustments in the muscle weights of the vastus lateralis within a specific synergy pattern. Our findings substantiate our hypothesis that the quantity of muscle synergies did not differ between the groups; patients with CAI may adapt to biomechanical alterations following ankle sprains by rationalizing changes in muscle-specific weights across various motor modules.

An initial significant finding of this study was that individuals with CAI exhibited increased muscle-specific weights of the gluteus maximus in Synergy 1 compared to healthy groups. Synergy 1 corresponds to hip strategy during landing, and the present findings support prior research indicating altered proximal hip biomechanics observed during landing in individuals with CAI [43]. The prevailing consensus in the existing literature suggests that reduced postural stability of the ankle joint among individuals with CAI leads to compromised ankle function. Consequently, CAI patients necessitate enhanced mechanisms of hip joint regulation to compensate for the deficiency in ankle function, facilitating the maintenance of static body balance [44,45,46]. This study utilized a matrix decomposition algorithm, further emphasizing the importance of the gluteus maximus in neuromotor control among patients with CAI. This observation highlights a pertinent clinical consideration that individuals with CAI exhibit a shift in movement patterns from distal to proximal limbs, emphasizing the need for robust strength and stability in the muscles surrounding the hip joint to accommodate the increased load. Indeed, alterations in the muscle-specific weight of the gluteus maximus were not only observed in Synergy 1, while in Synergy 2 we also found a slight adjustment. Despite the relatively small magnitude of muscle weights attributed to gluteus maximus in Synergy 2, potentially lacking a significant impact on muscle coordination function or clinical relevance, this finding underscores the pivotal role of the gluteus maximus in the comprehensive engagement of neuromuscular coordination patterns during landing in individuals with CAI.

Another significant observation is the impaired knee control strategies exhibited by CAI patients, characterized by decreased muscle activation levels. These alterations may be attributed to modified patterns of muscle recruitment within the vastus lateralis and the medial gastrocnemius. Notably, the ankle joint serves as the primary joint for impact absorption during landing [47,48,49]. However, an ankle sprain compromises the surrounding muscle groups’ capacity to adequately absorb ground impact, potentially increasing loads on other joints or transmitting impact forces to proximal static structures, thereby elevating the risk of proximal joint injury [50]. Several studies have confirmed the potential for knee injury during landing in patients with CAI. For instance, Gribble et al. observed a reduced knee flexion angle during landing prior to foot–ground contact in patients with CAI compared to controls [51]. This finding is consistent with the results reported by Terada et al., who demonstrated diminished knee cushioning capacity in CAI patients relative to healthy controls, along with a significant decrease in knee flexion angle at peak anterior tibial shear force [52]. Moreover, Kramer et al. observed a correlation between anterior cruciate ligament (ACL) injury occurrence and prior ankle sprains among athletes in their investigation of ACL injury risk factors [53]. Therefore, it is unsurprising that our study revealed altered spatiotemporal modules of knee-related neuromuscular coordination in individuals with CAI. Plausible speculation suggests that the impaired ankle joint control and recurrent sprains characteristic of CAI patients exacerbate proprioceptive abnormalities. This, in turn, hinders the effective activation of diverse muscle groups involved in lower limb activities, impacting muscle excitability and sensitivity, potentially resulting in an aberrant or uneven pattern of muscle activation. Therefore, forthcoming treatment strategies should integrate evaluations of knee stability and injury susceptibility in individuals with CAI, alongside comprehensive functional assessments focusing on the coordinated interplay of the vastus lateralis and lateral gastrocnemius. Further insights obtained from this study indicated increased involvement of the tibialis anterior during the ankle control strategy in CAI patients. This observation is in accordance with prior research emphasizing the crucial role of tibialis anterior function in compensating for deficits linked to ankle sprains, as demonstrated by Kim et al. in their exploration of lower limb muscle synergies during anticipated and unanticipated landing–cutting [27]. Some researchers suggests that the dorsiflexion posture of the ankle may represent a self-protective mechanism in individuals with CAI, who proactively dorsiflex the ankle prior to landing to achieve a more stable dorsiflexed posture and mitigate the risk of ligament re-sprains [15].

An especially interesting finding of the current study pertains to the observed alteration in Synergy 4 associated with Synergy 2, wherein adjustments were noted in the muscle weights of the vastus lateralis, peroneus longus, and lateral gastrocnemius. Synergy 4 involves a comprehensive, multi-joint motor control pattern, necessitating increased recruitment of multiple muscles to synchronize effectively. Consequently, it is presumed to play a significant role in the maintenance of overall body balance. Initial observations indicated a reduced contribution of the lateral gastrocnemius in Synergy 4 compared to healthy individuals, consistent with findings in Synergy 2. This phenomenon may be associated with the observed impaired knee control strategy. However, such a discrepancy may compromise overall body postural stability, given previous research indicating that increased activation of the lateral gastrocnemius, as a bi-articular muscle, facilitates coordinated enhancement of the co-activation pattern, thereby enhancing stability during landing [54]. Additionally, a notable decrease in the muscle weight of the peroneus longus was observed in patients with CAI. This reduction may contribute to an impaired ability to withstand the sudden increase in lateral foot loading during landing, potentially leading to a lateral shift in the plantar center of pressure and an elevated risk of ankle instability or sprains [55]. Given the observed variation in muscle weighting of the peroneus longus within a comprehensive lower limb control strategy, and recognizing its role as a key stabilizer of the ankle joint, should there be an emphasis on integrating a coordinated multi-joint control training program into the rehabilitation protocols for the CAI patients? Nevertheless, CAI patients appear to exhibit adaptive responses, as evidenced by the slight adjustment of muscle-specific weights in the vastus lateralis. This muscle has greater robustness than other knee extensors, and their heightened activation can effectively counteract lateral forces during landing while also limiting excessive leg swing during lateral movements to maintain stability [56]. Our analysis revealed that individuals with CAI actively adapt to biomechanical alterations resulting from ankle sprains by coordinating the muscle weights of the vastus lateralis to meet the new demands.

The current study has several limitations. Firstly, while examining muscle synergy patterns during landing is crucial for understanding movement in patients with CAI, focusing solely on this aspect may provide only a partial insight into the motor control strategies utilized by CAI patients. Exploring other facets of movement, such as gait, balance, or dynamic movements, would offer a more holistic understanding of motor control adaptations in individuals with CAI. Secondly, although our study included 22 patients with CAI and 22 healthy participants, the sample size, although potentially adequate for certain analyses, may benefit from larger cohorts to enhance statistical power and the generalizability of findings. Future investigations would benefit from expanding sample sizes to bolster the statistical robustness and reliability of results. Thirdly, while our methodology for extracting muscle synergies proved reliable and yielded significant findings, the unique characteristics of CAI patients, including patient-specific movement patterns and neuromuscular control strategies, raise the possibility that our findings may not be readily generalizable to other populations and exercise contexts. Fourthly, although efforts were made to minimize noise effects on surface EMG signal extraction through a self-built muscle activation model, the proximity of recorded muscles may have led to sensor crosstalk, potentially impacting EMG signal quality. Lastly, the absence of kinematic and kinetic data recording and interpretation in this study may have limited the comprehensive analysis of muscle synergy and its clinical implications.

## 5. Practical Applications

In light of the current study results, we propose several recommendations that may be beneficial for the implementation of rehabilitation plans: (1) It is essential to focus on rehabilitation interventions targeting the muscles around the knee joint to restore normal knee joint control strategies, with particular emphasis on exercises targeting the vastus lateralis, and medial gastrocnemius muscles. (2) From a muscle synergy perspective, rehabilitation training plans should not solely focus on isolated strengthening of individual muscles such as the tibialis anterior and peroneus longus, as highlighted in previous studies. Instead, emphasis should be placed on implementing rehabilitation training plans that promote multi-muscle coordination through specialized exercises. (3) The crucial role of the gluteus maximus in multiple movement modules (muscle synergy 1 and muscle synergy 2) underscores its importance, potentially stemming from compensatory mechanisms due to ankle joint movement deficits in CAI patients. Therefore, strengthening exercises targeting the gluteus maximus should be emphasized to adapt to increased loads.

## 6. Conclusions

The primary objective of this study was to explore muscle synergy patterns during landing among individuals with CAI. We first employed the NNMF algorithm to decompose muscle activations processed via a second-order discrete linear filter model to obtain muscle synergy patterns, and then classified using the K-means clustering algorithm and Pearson’s correlation coefficient to identify various neuromuscular organization strategies in CAI patients during landing. Our findings suggest that CAI patients may exhibit adaptive responses to biomechanical alterations following ankle sprains by modulating the temporal and spatial components of motor modules, particularly in coordinating the activities of the gluteus maximus and tibialis anterior. Notably, while deficiencies in the knee control module were observed in CAI, the unexpected identification of adjustment strategies involving the vastus lateralis across different modules sheds light on a new rehabilitative treatment for patients with CAI. Future studies should expand their scope to include a broader range of motor tasks to provide a more comprehensive understanding of motor control adaptations in patients with CAI.

## Figures and Tables

**Table 1:** Participant demographics.

	CAI (*n* = 22) Mean (SD)	Healthy (*n* = 22) Mean (SD)	*p*-Value
Age (year)	23.5 (1.2)	22.7 (1.4)	0.349
Mass (kg)	80.4 (5.6)	80.6 (6.1)	0.864
Height (cm)	179.7 (4.5)	178.8 (4.0)	0.643
CAIT (score)	20.9 (2.6)	29.5 (0.5)	<0.001
Ankle sprains (times)	3.2 (1.1)	0	<0.001

Note: SD: standard deviation; CAI: chronic ankle instability; CAIT: Cumberland Ankle Instability Tool.

**Table 2:** Descriptive data of the number of muscle synergies, global VAF, and local VAFs for each group.

	CAI Mean (SD)	Healthy Mean (SD)	*p*-Value
**The number of muscle synergies**	4.55 (0.58)	4.27 (0.45)	0.109
**Global VAF (%)**			
With three synergies	85.30 (2.13)	86.24 (1.72)	0.122
With four synergies	90.09 (1.61)	90.64 (1.23)	0.221
With five synergies	92.58 (1.10)	93.37 (1.12)	0.138
**Local VAFs with four synergies (%)**			
BF	88.62 (1.69)	84.19 (9.42)	0.297
GM	84.89 (4.44)	85.75 (4.33)	0.730
RF	90.27 (5.11)	94.18 (3.21)	0.128
VM	91.76 (3.11)	90.40 (3.47)	0.471
VL	89.74 (2.06)	88.40 (3.67)	0.426
MG	92.54 (2.02)	93.34 (2.58)	0.543
LG	90.81 (2.57)	91.84 (2.13)	0.452
SL	89.75 (3.92)	90.36 (0.90)	0.713
TA	88.05 (3.59)	87.10 (3.95)	0.658
PL	90.07 (5.03)	91.11 (1.83)	0.639

**Table 3:** Comparison of the synergy vectors of 10 muscles during landing between the CAI and healthy groups.

Synergy Vectors Mean (SD)	Muscle Synergy 1			Muscle Synergy 2			Muscle Synergy 3			Muscle Synergy 4		
	CAI	Healthy	*p*-Value	CAI	Healthy	*p*-Value	CAI	Healthy	*p*-Value	CAI	Healthy	*p*-Value
$I_{BF}$	0.71 (0.14)	0.68 (0.15)	0.586	0.03 (0.07)	0.04 (0.05)	0.804	0.28 (0.20)	0.13 (0.11)	0.026	0.08 (0.07)	0.22 (0.15)	0.002
$I_{GM}$	0.50 (0.25)	0.32 (0.13)	**0.035**	0.13 (0.18)	0.02 (0.03)	0.042	0.16 (0.11)	0.11 (0.07)	0.095	0.37 (0.29)	0.43 (0.19)	0.411
$I_{RF}$	0.11 (0.11)	0.07 (0.10)	0.698	0.90 (0.13)	0.92 (0.17)	0.635	0.10 (0.10)	0.13 (0.12)	0.454	0.49 (0.22)	0.52 (0.35)	0.766
$I_{VM}$	0.11 (0.10)	0.12 (0.11)	0.835	0.39 (0.22)	0.43 (0.23)	0.642	0.16 (0.13)	0.12 (0.11)	0.335	0.54 (0.28)	0.64 (0.21)	0.324
$I_{VL}$	0.24 (0.20)	0.22 (0.14)	0.785	0.30 (0.20)	0.46 (0.20)	0.018	0.13 (0.09)	0.12 (0.11)	0.894	0.45 (0.17)	0.31 (0.15)	0.030
$I_{MG}$	0.27 (0.18)	0.25 (0.15)	0.763	0.41 (0.18)	0.43 (0.26)	0.730	0.69 (0.31)	0.74 (0.27)	0.619	0.18 (0.17)	0.19 (0.15)	0.922
$I_{LG}$	0.25 (0.19)	0.23 (0.12)	0.749	0.30 (0.18)	0.43 (0.21)	0.049	0.47 (0.20)	0.41 (0.23)	0.383	0.30 (0.14)	0.52 (0.20)	0.005
$I_{SL}$	0.10 (0.10)	0.09 (0.05)	0.810	0.40 (0.26)	0.44 (0.18)	0.482	0.20 (0.15)	0.11 (0.07)	0.065	0.54 (0.14)	0.58 (0.16)	0.599
$I_{TA}$	0.25 (0.16)	0.26 (0.12)	0.813	0.09 (0.08)	0.13 (0.10)	0.163	0.64 (0.31)	0.48 (0.22)	0.042	0.08 (0.07)	0.04 (0.06)	0.186
	0.13 (0.10)	0.11 (0.09)	0.714	0.29 (0.20)	0.14 (0.12)	0.006	0.21 (0.14)	0.24 (0.23)	0.645	0.31 (0.20)	0.53 (0.30)	0.047

Note: CAI: chronic ankle instability; SD: standard deviation.

## Data Availability

The data are not publicly available due to privacy or ethical restrictions.

## Supplementary Materials

The following supporting information can be downloaded at: https://www.mdpi.com/article/10.3390/bioengineering11050518/s1, Supplementary Text S1. Maximal Voluntary Contraction Acquisition Procedure. Supplementary Text S2. Synergy Vectors and Activation Coefficients Figures. Supplementary Text S3. Correlation Coefficients Figures. Supplementary Text S4. The CAIT Questionnaire.

## References

[1] Hølmer P., Søndergaard L., Konradsen L., Nielsen P.T., Jørgensen L.N. (1994). Epidemiology of sprains in the lateral ankle and foot. Foot Ankle Int..

[2] Wikstrom E.A., Hubbard-Turner T., McKeon P.O. (2013). Understanding and treating lateral ankle sprains and their consequences: A constraints-based approach. Sports Med..

[3] Gribble P.A., Bleakley C.M., Caulfield B.M., Docherty C.L., Fourchet F., Fong D.T.-P., Hertel J., Hiller C.E., Kaminski T.W., McKeon P.O. (2016). Evidence review for the 2016 International Ankle Consortium consensus statement on the prevalence, impact and long-term consequences of lateral ankle sprains. Br. J. Sports Med..

[4] Yu P., Mei Q., Xiang L., Fernandez J., Gu Y. (2022). Differences in the locomotion biomechanics and dynamic postural control between individuals with chronic ankle instability and copers: A systematic review. Sports Biomech..

[5] Wikstrom E.A., Brown C.N. (2014). Minimum reporting standards for copers in chronic ankle instability research. Sports Med..

[6] Thompson C., Schabrun S., Romero R., Bialocerkowski A., van Dieen J., Marshall P. (2018). Factors contributing to chronic ankle instability: A systematic review and meta-analysis of systematic reviews. Sports Med..

[7] Li H.-Y., Zheng J.-J., Zhang J., Cai Y.-H., Hua Y.-H., Chen S.-Y. (2016). The improvement of postural control in patients with mechanical ankle instability after lateral ankle ligaments reconstruction. Knee Surg. Sports Traumatol. Arthrosc..

[8] Needle A.R., Lepley A.S., Grooms D.R. (2017). Central nervous system adaptation after ligamentous injury: A summary of theories, evidence, and clinical interpretation. Sports Med..

[9] Xu D., Quan W., Zhou H., Sun D., Baker J.S., Gu Y. (2022). Explaining the differences of gait patterns between high and low-mileage runners with machine learning. Sci. Rep..

[10] Tresch M.C., Saltiel P., Bizzi E. (1999). The construction of movement by the spinal cord. Nat. Neurosci..

[11] Gao X., Xu D., Li F., Baker J.S., Li J., Gu Y. (2023). Biomechanical Analysis of Latin Dancers’ Lower Limb during Normal Walking. Bioengineering.

[12] Clark D.J., Ting L.H., Zajac F.E., Neptune R.R., Kautz S.A. (2010). Merging of healthy motor modules predicts reduced locomotor performance and muscle coordination complexity post-stroke. J. Neurophysiol..

[13] Lin J.-Z., Lin Y.-A., Lee H.-J. (2019). Are landing biomechanics altered in elite athletes with chronic ankle instability. J. Sports Sci. Med..

[14] Son S.J., Kim H., Seeley M.K., Hopkins J.T. (2017). Movement Strategies among Groups of Chronic Ankle Instability, Coper, and Control. Med. Sci. Sports Exerc..

[15] Wright C.J., Arnold B.L., Ross S.E. (2016). Altered kinematics and time to stabilization during drop-jump landings in individuals with or without functional ankle instability. J. Athl. Train..

[16] Doherty C., Bleakley C., Hertel J., Caulfield B., Ryan J., Delahunt E. (2016). Single-leg drop landing movement strategies in participants with chronic ankle instability compared with lateral ankle sprain ‘copers’. Knee Surg. Sports Traumatol. Arthrosc..

[17] Chen G., Han Y., Li Y., Shen J., Tu J., Yu Z., Zhang J., Cheng H., Zhu L., Dong F. (2024). Autonomous gait switching method and experiments of a hexapod walking robot for Mars environment with multiple terrains. Intell. Serv. Robot..

[18] Hubbard-Turner T., Turner M.J. (2015). Physical activity levels in college students with chronic ankle instability. J. Athl. Train..

[19] Dorris H., Oh J., Jacobson N. (2024). Wearable Movement Data as a Potential Digital Biomarker for Chronic Pain: An Investigation Using Deep Learning. Phys. Act. Health.

[20] Saito H., Yokoyama H., Sasaki A., Nakazawa K. (2023). Muscle synergy patterns as altered coordination strategies in individuals with chronic low back pain: A cross-sectional study. J. Neuroeng. Rehabil..

[21] Kerkman J.N., Daffertshofer A., Gollo L.L., Breakspear M., Boonstra T.W. (2018). Network structure of the human musculoskeletal system shapes neural interactions on multiple time scales. Sci. Adv..

[22] Oliveira A.S., Silva P.B., Lund M.E., Kersting U.G., Farina D. (2013). Fast changes in direction during human locomotion are executed by impulsive activation of motor modules. Neuroscience.

[23] d’Avella A., Saltiel P., Bizzi E. (2003). Combinations of muscle synergies in the construction of a natural motor behavior. Nat. Neurosci..

[24] Cheung V.C., d’Avella A., Tresch M.C., Bizzi E. (2005). Central and sensory contributions to the activation and organization of muscle synergies during natural motor behaviors. J. Neurosci..

[25] Lee D.D., Seung H.S. (1999). Learning the parts of objects by non-negative matrix factorization. Nature.

[26] Rabbi M.F., Pizzolato C., Lloyd D.G., Carty C.P., Devaprakash D., Diamond L.E. (2020). Non-negative matrix factorisation is the most appropriate method for extraction of muscle synergies in walking and running. Sci. Rep..

[27] Kim H., Palmieri-Smith R., Kipp K. (2023). Muscle synergies in people with chronic ankle instability during anticipated and unanticipated landing-cutting tasks. J. Athl. Train..

[28] Fong D.T.-P., Hong Y., Chan L.-K., Yung P.S.-H., Chan K.-M. (2007). A systematic review on ankle injury and ankle sprain in sports. Sports Med..

[29] Li F., Xu D., Zhou H., Kovács B., Liang M. (2024). The effect of heel height on the Achilles tendon and muscle activity in Latin dancers during a special-landing task. Int. J. Biomed. Eng. Technol..

[30] Herzog W., Sokolosky J., Zhang Y., Guimarães A. (1998). EMG-force relation in dynamically contracting cat plantaris muscle. J. Electromyogr. Kinesiol..

[31] Van Ruijven L., Weijs W. (1990). A new model for calculating muscle forces from electromyograms. Eur. J. Appl. Physiol. Occup. Physiol..

[32] Buchanan T.S., Lloyd D.G., Manal K., Besier T.F. (2004). Neuromusculoskeletal modeling: Estimation of muscle forces and joint moments and movements from measurements of neural command. J. Appl. Biomech..

[33] Xu D., Zhou H., Quan W., Gusztav F., Baker J.S., Gu Y. (2023). Adaptive neuro-fuzzy inference system model driven by the non-negative matrix factorization-extracted muscle synergy patterns to estimate lower limb joint movements. Comput. Methods Programs Biomed..

[34] Xu D., Zhou H., Quan W., Gusztav F., Wang M., Baker J.S., Gu Y. (2023). Accurately and effectively predict the ACL force: Utilizing biomechanical landing pattern before and after-fatigue. Comput. Methods Programs Biomed..

[35] Berry M.W., Browne M., Langville A.N., Pauca V.P., Plemmons R.J. (2007). Algorithms and applications for approximate nonnegative matrix factorization. Comput. Stat. Data Anal..

[36] Lee D., Seung H.S. (2000). Algorithms for Non-Negative Matrix factorization. Adv. Neural Inf. Process. Syst..

[37] Danion F., Latash M. (2010). Motor Control: Theories, Experiments, and Applications.

[38] Li X., Zeng H., Li Y., Song A. (2023). Quantitative Assessment via Multi-Domain Fusion of Muscle Synergy Associated with Upper-Limb Motor Function for Stroke Rehabilitation. IEEE Trans. Biomed. Eng..

[39] Krishna K., Murty M.N. (1999). Genetic K-means algorithm. IEEE Trans. Syst. Man Cybern. Part B (Cybern.).

[40] Modha D.S., Spangler W.S. (2003). Feature weighting in k-means clustering. Mach. Learn..

[41] Choi Y., Kim Y., Kim M., Yoon B. (2019). Muscle synergies for turning during human walking. J. Mot. Behav..

[42] Pataky T.C. (2012). One-dimensional statistical parametric mapping in Python. Comput. Methods Biomech. Biomed. Eng..

[43] Kim H., Palmieri-Smith R., Kipp K. (2022). Peak forces and force generating capacities of lower extremity muscles during dynamic tasks in people with and without chronic ankle instability. Sports Biomech..

[44] McCann R.S., Crossett I.D., Terada M., Kosik K.B., Bolding B.A., Gribble P.A. (2017). Hip strength and star excursion balance test deficits of patients with chronic ankle instability. J. Sci. Med. Sport.

[45] Yen S.-C., Chui K.K., Corkery M.B., Allen E.A., Cloonan C.M. (2017). Hip-ankle coordination during gait in individuals with chronic ankle instability. Gait Posture.

[46] Robbins S., Waked E. (1998). Factors associated with ankle injuries: Preventive measures. Sports Med..

[47] Yeow C.H., Lee P.V.S., Goh J.C.H. (2011). An investigation of lower extremity energy dissipation strategies during single-leg and double-leg landing based on sagittal and frontal plane biomechanics. Hum. Mov. Sci..

[48] Xu D., Zhou H., Quan W., Jiang X., Liang M., Li S., Ugbolue U.C., Baker J.S., Gusztav F., Ma X. (2024). A new method proposed for realizing human gait pattern recognition: Inspirations for the application of sports and clinical gait analysis. Gait Posture.

[49] Xu D., Zhou H., Quan W., Ma X., Chon T.-E., Fernandez J., Gusztav F., Kovács A., Baker J.S., Gu Y. (2024). New insights optimize landing strategies to reduce lower limb injury risk. Cyborg Bionic Syst..

[50] Kim H., Son S.J., Seeley M.K., Hopkins J.T. (2018). Kinetic Compensations due to Chronic Ankle Instability during Landing and Jumping. Med. Sci. Sports Exerc..

[51] Gribble P., Robinson R. (2010). Differences in spatiotemporal landing variables during a dynamic stability task in subjects with CAI. Scand. J. Med. Sci. Sports.

[52] Terada M., Pfile K.R., Pietrosimone B.G., Gribble P.A. (2013). Effects of chronic ankle instability on energy dissipation in the lower extremity. Med. Sci. Sports Exerc..

[53] Kramer L., Denegar C., Buckley W., Hertel J. (2007). Factors associated with anterior cruciate ligament injury: History in female athletes. J. Sports Med. Phys. Fit..

[54] DeMers M.S., Hicks J.L., Delp S.L. (2017). Preparatory co-activation of the ankle muscles may prevent ankle inversion injuries. J. Biomech..

[55] Nyska M., Shabat S., Simkin A., Neeb M., Matan Y., Mann G. (2003). Dynamic force distribution during level walking under the feet of patients with chronic ankle instability. Br. J. Sports Med..

[56] Madigan M.L., Pidcoe P.E. (2003). Changes in landing biomechanics during a fatiguing landing activity. J. Electromyogr. Kinesiol..

