# Supplementary material for: Structural and Organizational Strategies of Locomotor Modules during Landing in Patients with Chronic Ankle Instability
Source: Bioengineering (Basel). 2024 May 20;11(5):518. doi: 10.3390/bioengineering11050518 (PMC11117571; doi:10.3390/bioengineering11050518)
Supplement: Supplementary file 1 [file bioengineering-11-00518-s001.zip › bioengineering-3002220-supplementary.pdf]

Supporting Material

Structural and organizational strategies of locomotor modules during landing in patients with chronic ankle instability

This Supporting Material file includes:

1. Supplementary Text 1 - Maximal Voluntary Contraction Acquisition Procedure

2. Supplementary Text 2 - Synergy Vectors and Activation Coefficients Figures

3. Supplementary Text 3 - Correlation Coefficients Figures

4. Supplementary Text 4 - The CAIT Questionnaire

1. Supplementary Text 1 - Maximal Voluntary Contraction Acquisition Procedure

Participants underwent the MVC test while seated in the plyometric testing system. During the procedure, participants maintained a perpendicular torso position, with their eyes directed forward and hands resting naturally, while their waist was secured using a harness. For MVC testing of the rectus femoris, vastus medialis, and vastus lateralis muscles, participants were fixed at the base of the thigh and calf, and instructed to extend the knee with maximum force for 3-5 seconds. For the biceps femoris MVC, participants were fixed at the thigh and calf and asked to perform knee flexion with maximal force for 3-5 seconds. Similarly, for the gluteus maximus MVC test, participants were fixed at the thigh and calf and instructed to extend the hip with maximum force for 3-5 seconds. Testing the tibialis anterior and peroneus longus muscles involved fixing the thigh and foot, and participants were instructed to perform ankle dorsiflexion and eversion movements, respectively, at maximal force for 3-5 seconds. Lastly, for MVC testing of the medial and lateral gastrocnemius and the soleus muscle, participants' thighs and feet were fixed, and they were asked to perform ankle plantarflexion at maximum force for 3-5 seconds. A 20-second interval was observed between each test, with the maximum value recorded as the MVC for each muscle.

2. Supplementary Text 2 - Synergy Vectors and Activation Coefficients Figures.

Non-negative matrix decomposition was performed on muscle activation data to visually represent muscle synergy components. The decomposition results consist of synergy vectors and activation coefficients for each subject in both groups.

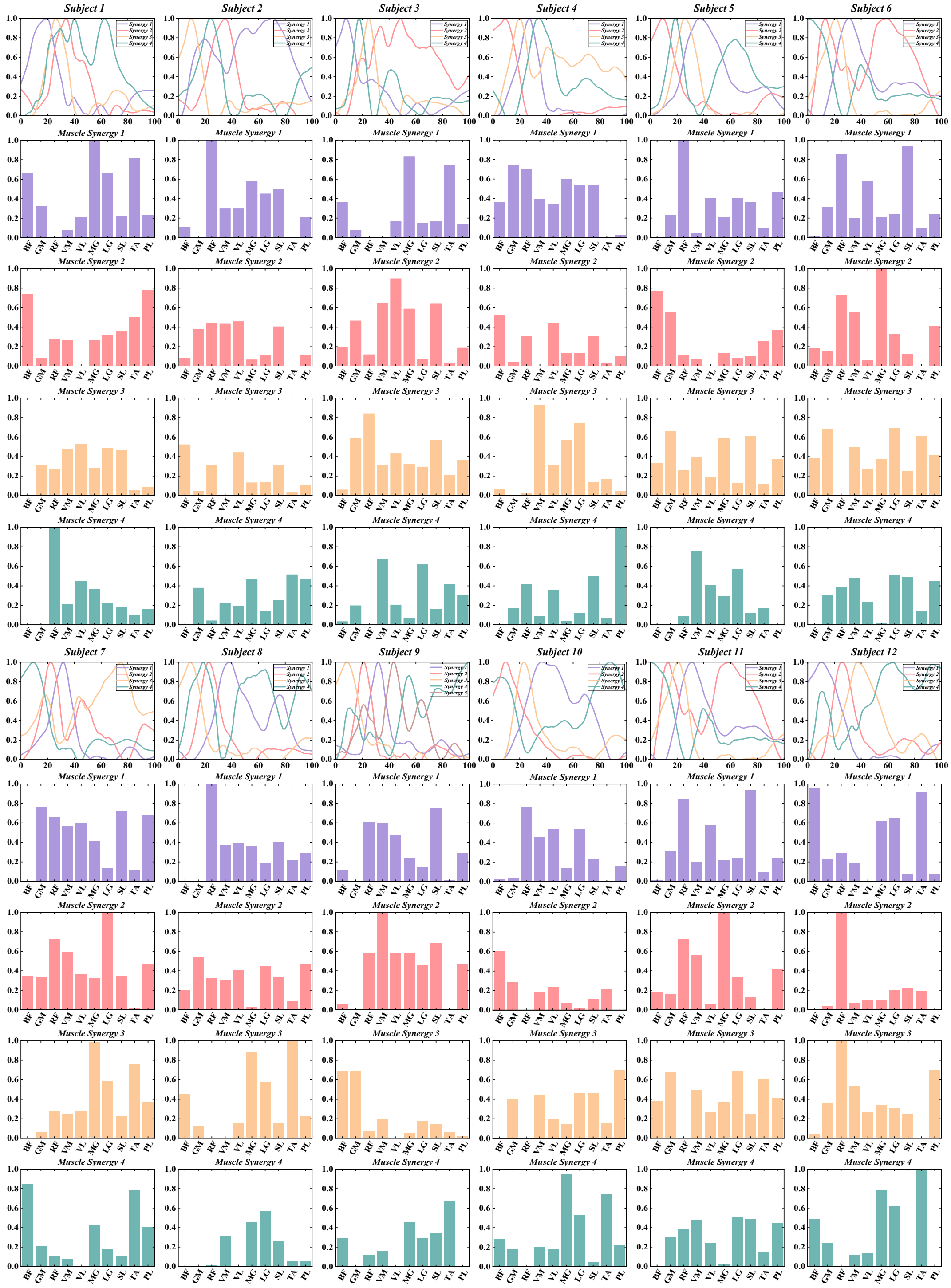

Figure S1. The extracted synergy vectors matrix and activation coefficient matrix from 1- 12 subjects in the CAI group are presented, with activation coefficient results displayed in rows 1 and 6, and the remaining rows depicting the synergy vectors.

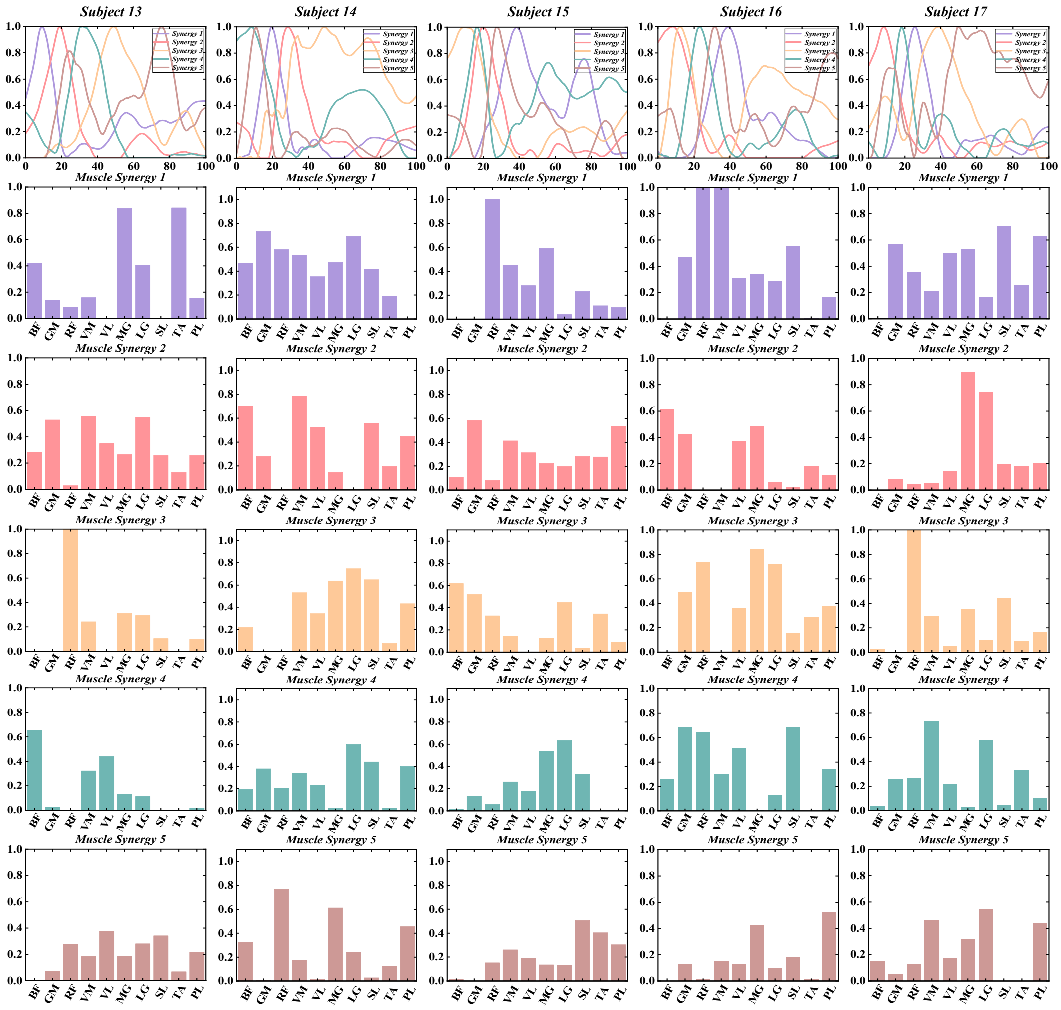

Figure S2. The extracted synergy vectors matrix and activation coefficient matrix from 13-17 subjects in the CAI group.

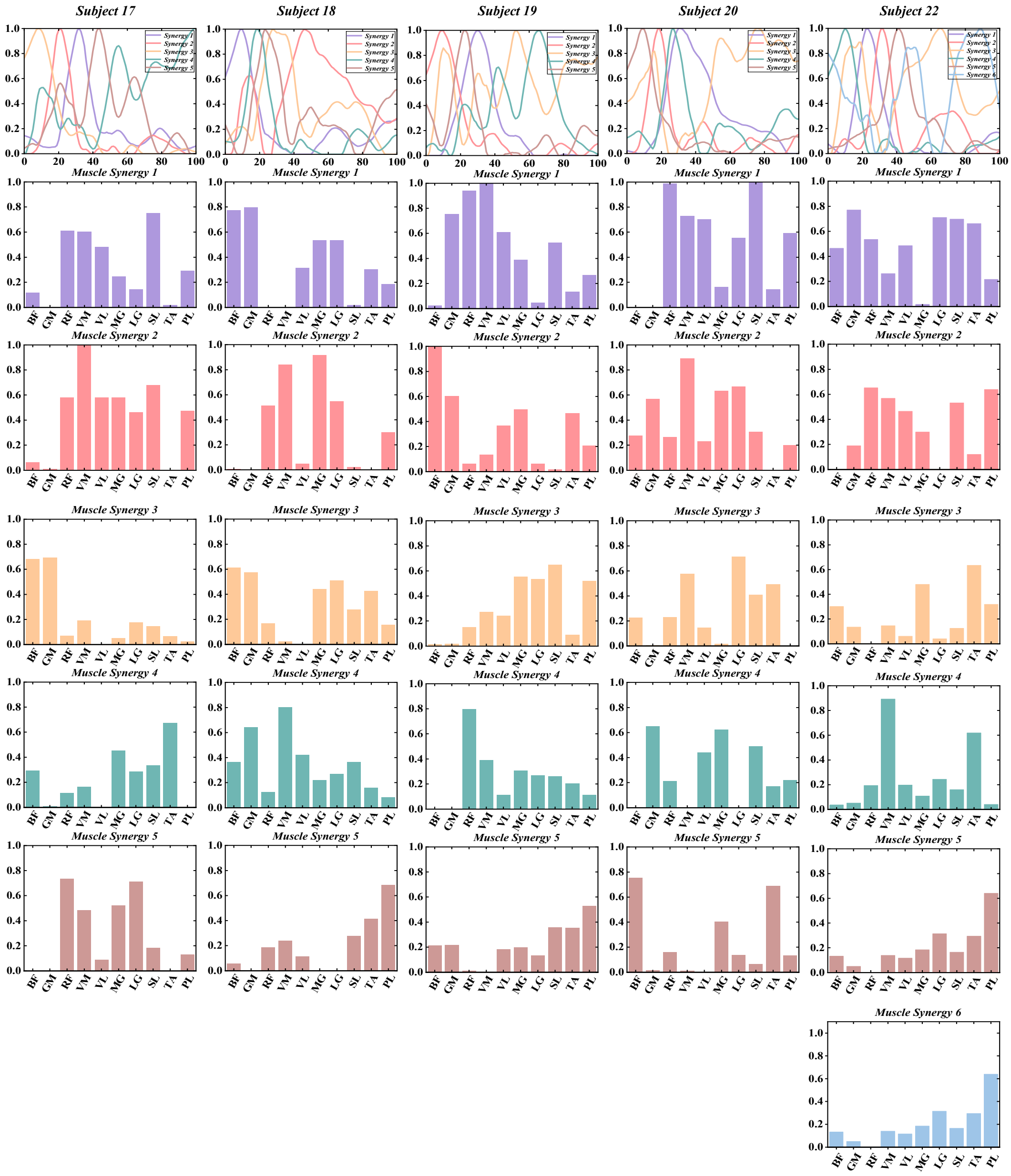

Figure S3. The extracted synergy vectors matrix and activation coefficient matrix from 18-22 subjects in the CAI group.

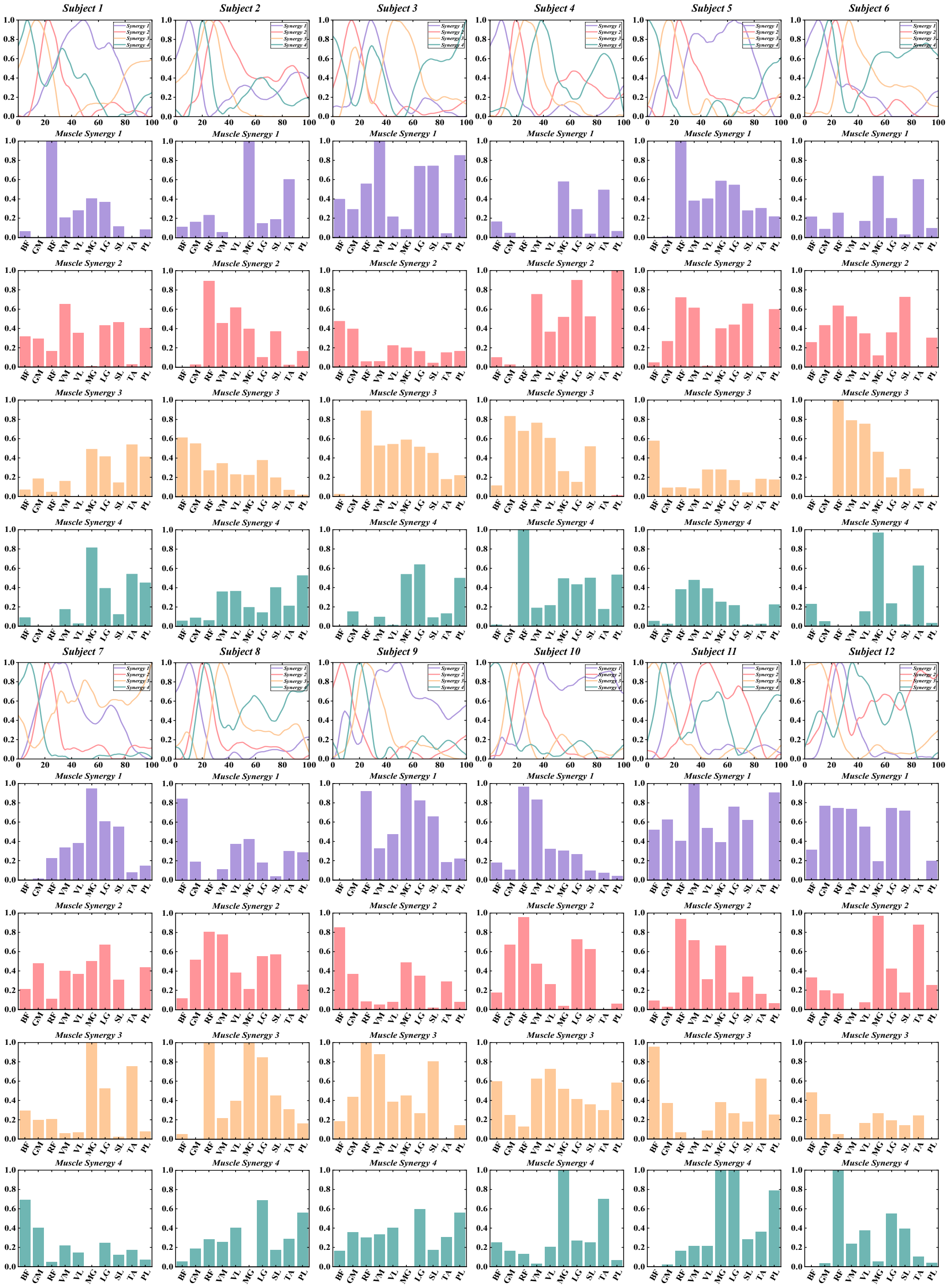

Figure S4. The extracted synergy vectors matrix and activation coefficient matrix from 1- 12 subjects in the healthy group are presented, with activation coefficient results displayed in rows 1 and 6, and the remaining rows depicting the synergy vectors.

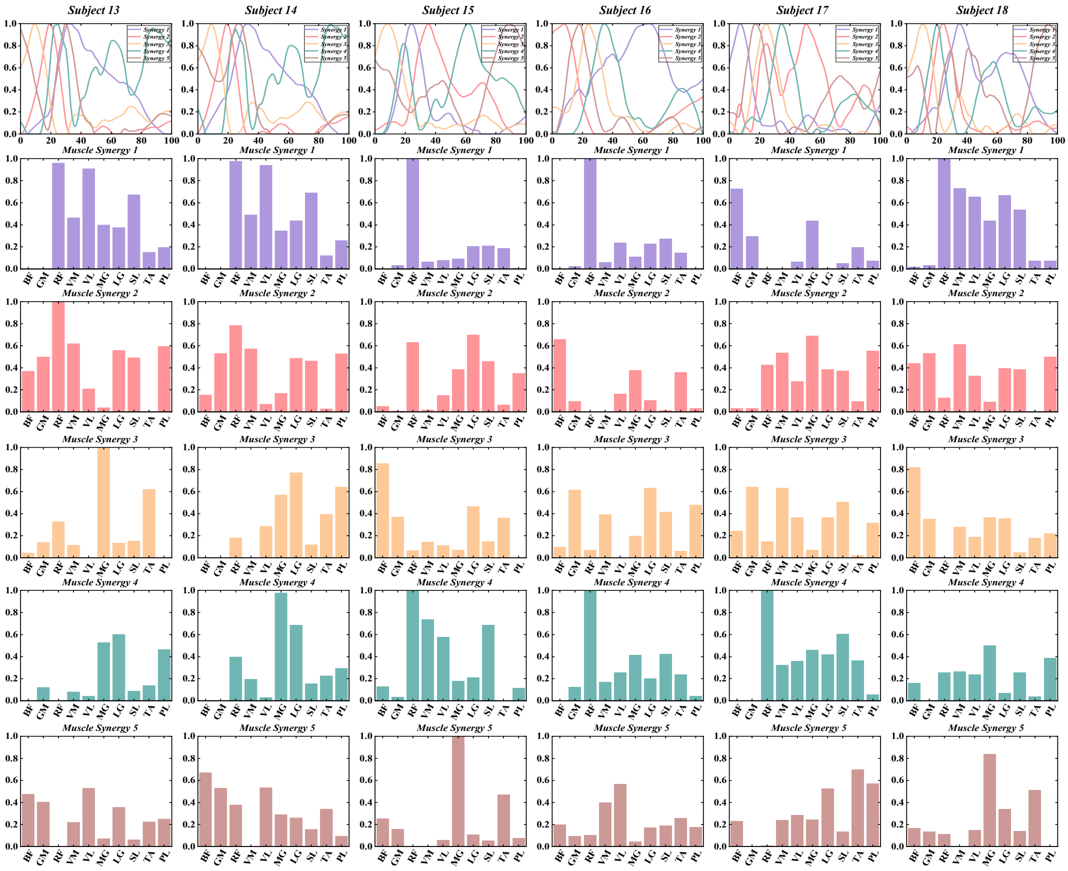

Figure S5. The extracted synergy vectors matrix and activation coefficient matrix from 13-18 subjects in the healthy group.

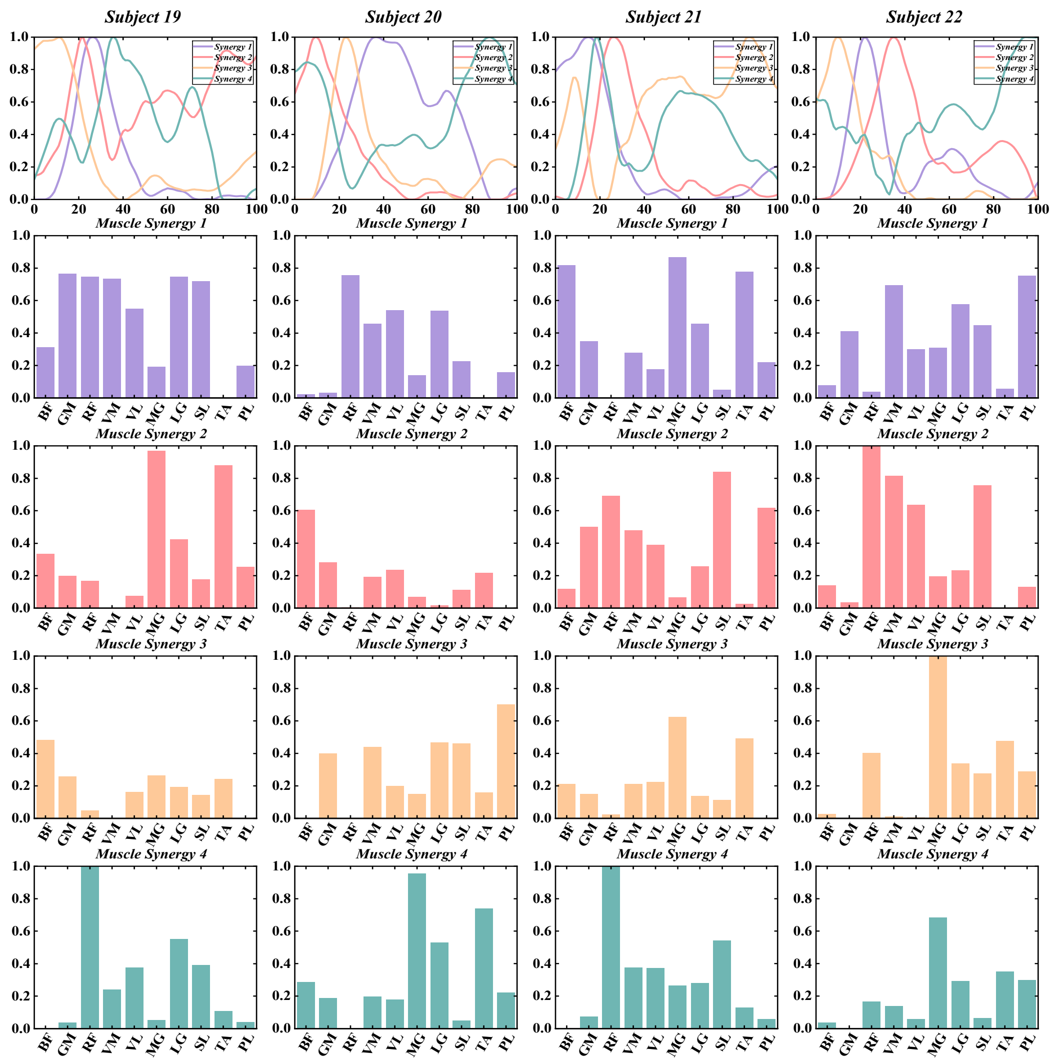

Figure S6. The extracted synergy vectors matrix and activation coefficient matrix from 19-22 subjects in the healthy group.

3. Supplementary Text 3 - Correlation coefficients Figures

The Pearson correlation coefficient was utilized to assess the correlation between the synergy vectors generated by the non-negative matrix factorization algorithm and the reference synergy across all subjects in both groups.

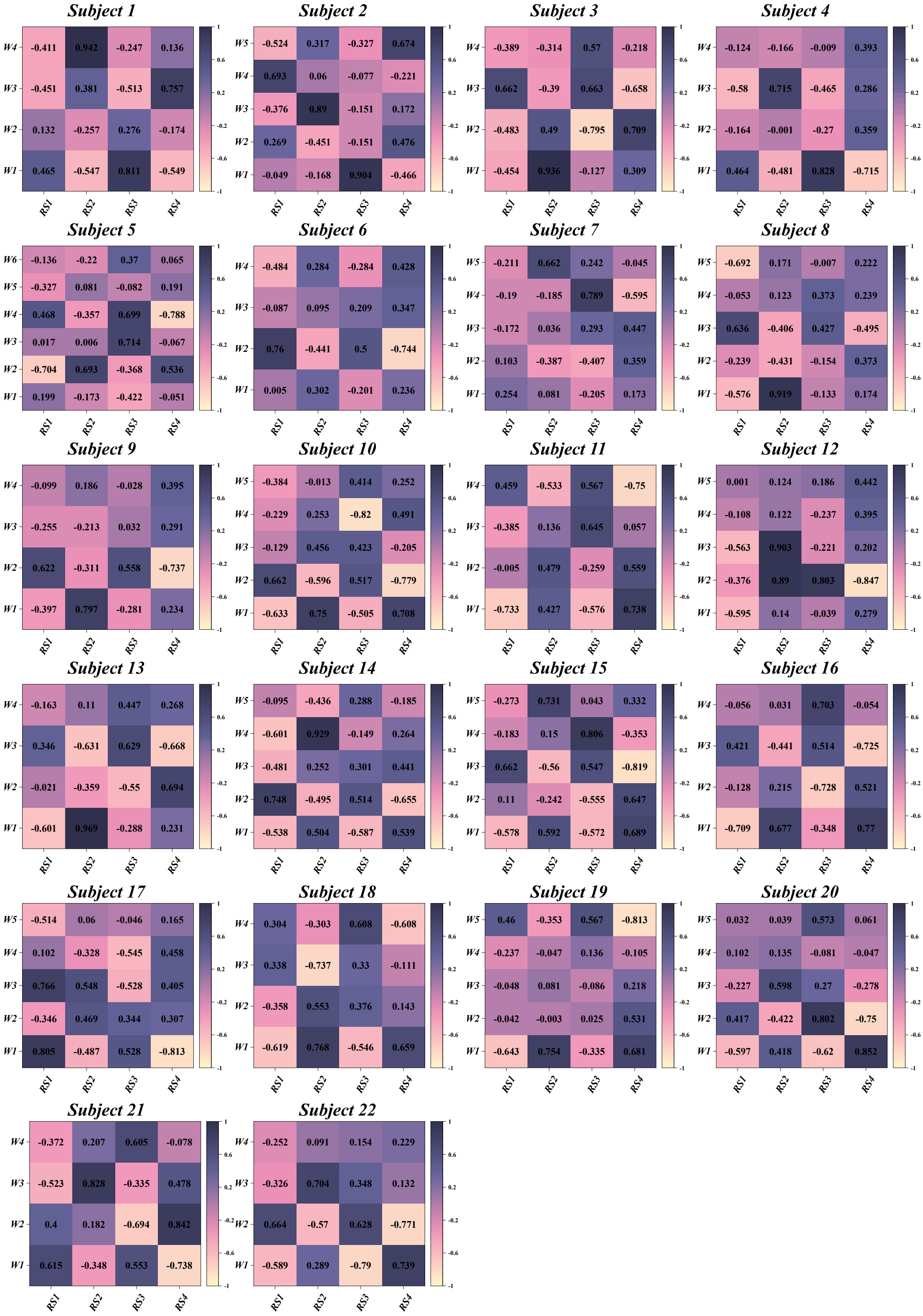

Figure S7. Correlation coefficients between synergy vectors and reference synergies for the 22 subjects in the CAI group. W: synergy vectors. RS: Reference Synergy

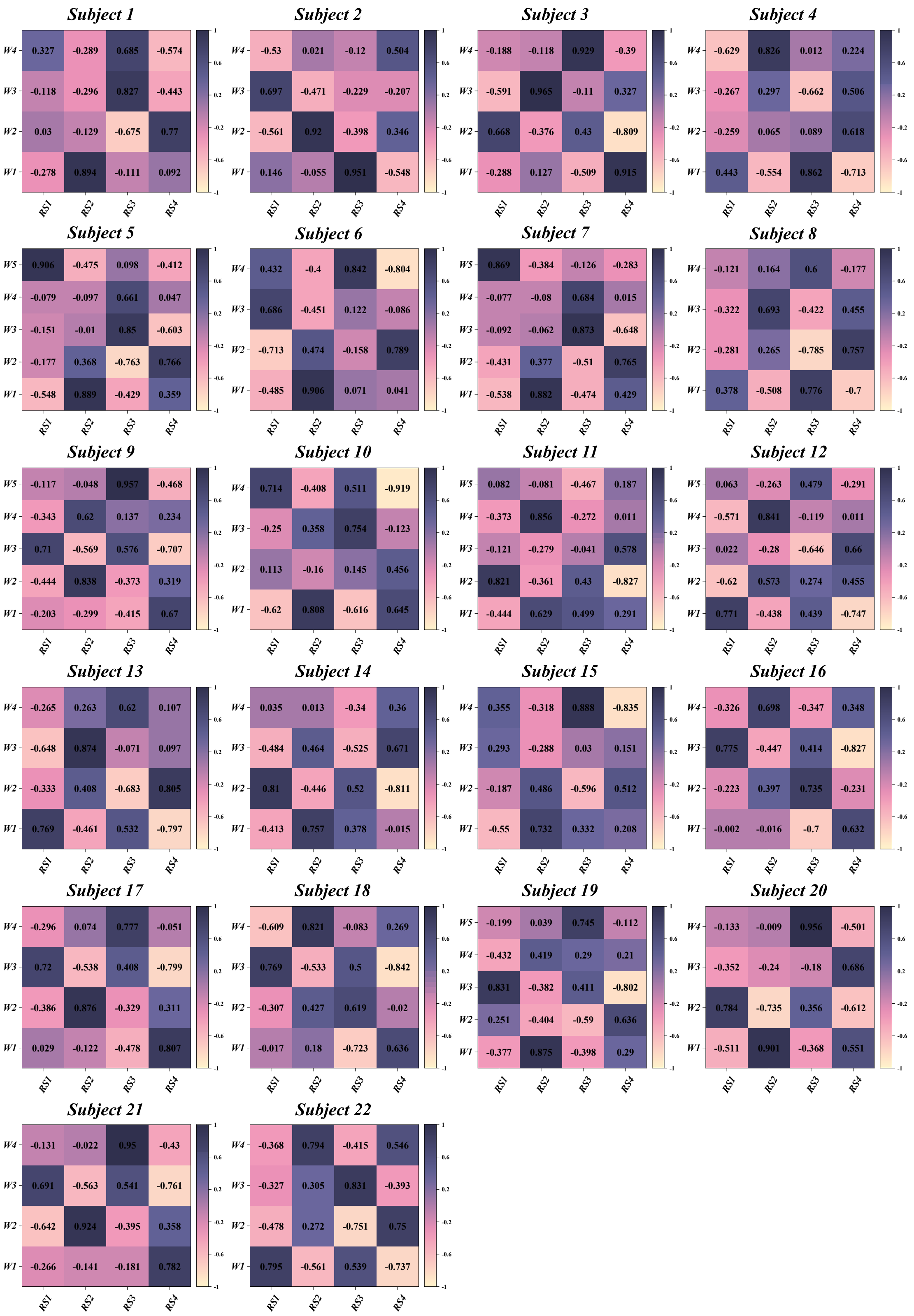

Figure S8. Correlation coefficients between synergy vectors and reference synergies for the 22 subjects in the healthy group.

4. Supplementary Text 4 - The CAIT Questionnaire

THE CAIT QUESTIONNAIRE

Please tick the ONE statement in EACH question that BEST describes your ankles.

	LEFT	RIGHT	Score
1.I have pain in my ankle			
Never			**5**
During sport			**4**
Running on uneven surfaces			**3**
Running on level surfaces			**2**
Walking on uneven surfaces			**1**
Walking on level surfaces			**0**
2.My ankle feels UNSTABLE			
Never			**4**
Sometimes during sport not every time)			**3**
Frequently during sport (every time)			**2**
Sometimes during daily activity			**1**
Frequently during daily activity			**0**
3. When l make SHARP turns, my ankle feels UNSTABLE			
Never			**3**
Sometimes when running			**2**
Often when running			**1**
When walking			**0**
4. When going down the stairs, my ankle feels UNSTABLE			
Never			**3**
If I go fast			**2**
Occasionally			**1**
Always			**0**
5. My ankle feels UNSTABLE when standing on ONE leg			
Never			**2**
On the ball of my foot			**1**
With my foot flat			**0**
6.My ankle feels UNSTABLE when			
Never			**3**
I hop from side to side			**2**
I hop on the spot			**1**
When l jump			**0**
7. My ankle feels UNSTABLE when			
Never			**4**
I run on uneven surfaces			**3**

I jog on uneven surfaces	**2**
I walk on uneven surfaces	**1**
I walk on a flat surface	**0**
8.TYPICALLY, when l start to roll over (or "twist") on my ankle, l can stop it	
Immediately	**3**
Often	**2**
Sometimes	**1**
Never	**0**
I have never rolled over on my ankle	**3**
9.After a TYPlCAL incident of my ankle rolling over, my ankle returns to "normal"	
Almost immediately	**3**
Less than one day	**2**
1-2 days	**1**
More than 2 days	**0**
I have never rolled over on my ankle	**3**

NOTE. The scoring scale is on the right. The scoring system is not visible on the subject's version.
